# Life histories in Fiji as reconstructed from first millennium CE Sigatoka Sand Dune burials using isotopes

**DOI:** 10.1371/journal.pone.0300749

**Published:** 2024-05-09

**Authors:** Estelle Herrscher, Frédérique Valentin, Wanda Zinger, Baptiste Pradier, Guy André

**Affiliations:** 1 Aix Marseille University, CNRS, Minist Culture, LAMPEA, Aix–en–Provence, France; 2 UMR 8068, TEMPS, MSH Mondes, Nanterre, France; 3 Institute for Archaeological Sciences, Archaeo–and Palaeogenetics Group, University of Tübingen, Tübingen, Germany; University of Bern: Universitat Bern, SWITZERLAND

## Abstract

This paper aims to re-examine the dietary practices of individuals buried at Sigatoka Sand Dunes site (Fiji) in Burial Ground 1 excavated by Simon Best in 1987 and 1988 using two approaches and a reassessment of their archaeological, bioarchaeological and chronological frame. First, stable carbon and nitrogen isotope analysis was applied to document dietary changes between childhood and adulthood using an intra–individual approach on paired bone–tooth. Second, the potential adaptation of the individuals to their environment was evaluated through regional and temporal comparisons using inter–individual bone analysis. Ten AMS radiocarbon dates were measured directly on human bone collagen samples, placing the series in a range of approximately 600 years covering the middle of the first millennium CE (1,888 to 1,272 cal BP). *δ*^13^C and *δ*^15^N ratios were measured on bone and tooth collagen samples from 38 adult individuals. The results show that *δ*^15^N values from tooth are higher than those s from bone while bone and tooth *δ*^13^C values are similar, except for females. Fifteen individuals were included in an intra–individual analysis based on paired bone and tooth samples, which revealed six dietary patterns distinguished by a differential dietary intake of marine resources and resources at different trophic levels. These highlight sex–specific differences not related to mortuary practices but to daily life activities, supporting the hypothesis of a sexual division of labour. Compared to other Southwest Pacific series, Sigatoka diets show a specific trend towards marine food consumption that supports the hypothesis of a relative food self–sufficiency requiring no interactions with other groups.

## Introduction

Stable isotope analysis is an approach that has been widely used in bioarchaeology for over 40 years, to characterise and understand the lifestyles of past societies. A wide range of issues is addressed, from the characteristics of the diet itself to an analysis of the socio-economic factors involved in managing and accessing food resources [1,2]. Carbon (^13^C/^12^C), nitrogen (^15^N/^14^N), and sulphur (^34^S/^32^S) isotopes are routinely used for diet reconstruction, while isotopes of oxygen (^18^O/^16^O), and radiogenic strontium (^87^Sr/^86^Sr) are used to evaluate the mobility of past populations. The interpretation of biochemical signatures obtained at inter-population, intra-population and intra-individual levels opens up robust perspectives for the study of past diet and mobility of human groups, allowing refinement of analyses and assessment of changes over the life course. Stable carbon isotope ratios (*δ*^13^C) are used to track consumers of food groups with specific values, more commonly consumers of C3 plants or C4 plants and consumers of marine or terrestrial resources [3]. Stable nitrogen isotope ratios (*δ*^15^N) are generally analysed to infer trophic positions of consumers [3,4].

In the Southwest Pacific area, subsistence strategies, economic systems and foodways are, together with patterns of human migration, interaction and diversification, among the main issues driving archaeological research in the region, from the earliest settlements, at least 50,000 years ago in Australia and northern Melanesia and around 3,500 years ago in southern Melanesia and Polynesia, to the historical period marked by the discoveries and the Western colonisation of the islands [5–7]. Introductions, translocations and domestication of animal and vegetal species in highly variable environments and ecologies as well as the ritual and subsistence use of terrestrial and marine resources, are at the centre of an intense debate [8–11] to which isotopic analyses of human remains contribute significantly since Ambrose’s pioneering work on the Marianas Islands [12]. The proximity of marine coastal and reef resources and a vegetation cover predominantly using a C3 photosynthetic pathway allow the use of stable carbon and nitrogen isotopic ratios to distinguish between the contribution of terrestrial and marine resources to human diets. The contrasting dietary patterns between these two types of food have been largely documented in the literature since the early 2000s with a wide range of human isotopic values comprised between –19.7 to –13.5 ‰ and from 7.3 to 16.1 ‰ for *δ*^13^C and *δ*^15^N, respectively [12–22].

Based mainly on bone collagen samples and documenting the average diet of individuals in the later part of their lives, these studies have drawn several lines of conclusions. Irrespective of geography and culture, as shown for the initial settlements of Teouma in Vanuatu and Chelechol ra Orrak in Palau, isotopic data converge to indicate that the first inhabitants of these regions had a food economy that included significant use of marine protein sources [17,23]. A diachronic change in diet has been observed in the Vanuatu archipelago between Lapita individuals (3,000–2,800 years ago), considered to be the initial occupation, and Post–Lapita individuals (2,500–2,000 years ago), representing more established settlements. They are characterised by a mixed marine and terrestrial diet and a more terrestrial and vegetarian diet, respectively [17,18,21]. Specific geographical effects on diet have also been observed in relation to the dynamics of the island–human ecosystem, especially in Fiji and Tonga [19,24,25]. The island native biomass and the human group are factors that have constrained human dietary choices between wild foods and horticultural food [19,24,26,27]. Although there is no consensus on trace elements analysis, a pioneering analysis by Visser [28] of 10 individuals from Sigatoka Sand Dune burials, in Fiji, suggested that females consumed more shellfish than males without compromising their health. While the isotopic study conducted by Leach et al. [29] on one individual excavated by Best is worthy of mention, other studies providing broader isotopic insights into the Sigatoka population have been revived by the Phaff’s Master Thesis [30] and two published studies documenting adult diet and mobility based on series of individuals excavated by both Best and Burley. These highlight a mixed diet with terrestrial and marine components [31] and the presence of both local/coastal and non–local/inland individuals, mainly associated with recent times (Vuda, c. 450–350 years ago) [32]. Interestingly, Phaff et al. [31] hypothesis of inter-island socio–economic networks to supplement the low local availability of terrestrial and marine resources seems in contradiction with the presence of local individuals during the Fijian Plainware period, as confirmed by Cheung et al. [32].

Skeletal tissues undergo different turnover of their mineral and organic fractions depending on their type. The variety of biochemical signatures recorded in skeletal tissues make it possible to trace changes over the life of an individual and to reconstruct individual bionarratives, such as subadult diets from adult remains. Teeth contain characteristics of the period of their formation, while bones constantly renew their biogeochemical characteristics throughout life. The intra–individual approach was first used to track mobility of Chumash individuals, Native Amerindians of the Pacific coast of California [33]. This approach was then applied in numerous studies, primarily focusing on weaning–breastfeeding modalities to identify the age of the dietary changes, mainly in European populations [34–37]. Since the 2010s, studies largely expanded to include microsequential analysis of dental tissues to more accurately document changes in isotopic signal record [38,39]. More recently, the number of analyses using multiple tissues and multiple proxies with different sampling strategies has increased significantly, with the aim of better defining biochemical changes over the lifespan [40–43].

Intra–individual isotopic analyses have already been conducted in the Southwest Pacific area, at Namu site (c. 440–150 cal BP) [44], Southeast Solomon Islands, using two different methodological approaches [45,46]. The first, based on a comparison between bone (n = 142) and tooth (n = 86, M1 third distal root) samples, suggested that (i) deceased adolescents (9–17 years) and juveniles (5–9 yrs) had consumed less animal protein than surviving male adults and (ii) juveniles (5–9 years) had consumed more terrestrial food and less marine food than surviving adults [45]. The authors emphasised the difficulty of disentangling the effects of feeding strategies and other physiological stressors on surviving individuals. The second analysis used incremental analysis of first molar dentine to more precisely describe the feeding habits during the first years of life of 20 individuals [46]. The authors found an isotopic pattern specific to adolescence, resulting from dietary and health factors, probably related to the inability of individuals to survive to adulthood [46]. Another study of 16 individuals from Tutuila, American Samoa (1,200–100 cal BP), combining bone collagen and dentinal samples taken sequentially, demonstrates dietary changes related to social transitions during life, with (i) greater consumption of marine proteins between the age of 2 and 10 years and (ii) a gender-specific dietary patterns, female ’marine’ and male ’terrestrial’, which disappears by the age of 20 years [47]. Inter–individual isotope analysis of 16 individuals (740–150 cal BP) from Bourewa site in Fiji revealed a diet based predominantly on low–trophic level marine proteins, such as resources from coastal and reef environments, while individuals were expected to have consumed terrestrial resources from horticulture [48]. Interestingly, intra–individual analysis of bone and dentin isotope ratios across these individuals also revealed lower *δ*^13^C and higher *δ*^15^N values during childhood (5–10 years) interpreted as a more terrestrial diet and a more inland lifestyle.

On one hand, differences in isotopic signatures between sexes and ages at death or features of graves and mortuary treatments have been demonstrated to be significant, denoting preferential food choices function of sociocultural status in some South West Pacific populations [12,16,46,49,50]. On the other hand, the ^15^N enrichment of tissues can also be interpreted in terms of health, as resulting from physiological stress [51–54]. Physiological stress, linked primarily to a diet, poor in quality and quantity, can induce chain reactions such as the recycling from amino–acids by the body itself, leading to ^15^N enrichment of tissues [54,55]. Changes in oral health, also correlated with diet and physiological conditions [56], are rarely explored in relation to isotopic signature [16], although they provide an estimation of the level of physiological stress experienced by individuals at different stages of their lives. We anticipate that comparisons of isotopic signatures between bones and teeth in individuals unaffected and affected by physiological stress should help to disentangle whether the change relates to diet or physiology. More specifically, we suggest that 1) an analysis of carbon isotope ratios combined with socio–cultural criteria will enable us to establish a link between variations in the consumption of terrestrial and marine elements and specific dietary habits over the life course; and 2) the observation of ^15^N enrichment between bone and dental tissues in both affected and unaffected individuals will enable us to rule out the hypothesis of physiological stress caused by an inadequate diet.

In this paper, we first propose a new interpretation of the dietary practices of the individuals buried at Sigatoka Sand Dune Burial Ground 1 [50,57], using isotopic data representing different life times of 30 individuals to highlight potential change that are not visible in adult bone alone. By including paired bone and teeth, we aim to determine whether or not individuals are affected by dietary or physiological changes between childhood and adult periods or not. Secondly, we propose an analysis of the interaction between global factors, highlighting the environmental and anthropic pressures affecting the Sigatoka individuals. To this end, we have performed a meta–isotopic analysis comparing Southwest Pacific series related to a period comprised between 3,000 to 1,300 BP, using our and literature data on bone collagen sample. Finally, we are also attempting to determine to what extent the dietary choices of individuals from Sigatoka Burial Ground 1 shed light on the local availability of resources, or even their self–sufficiency, given the low mobility highlighted for this group [32]. Such a multifaceted investigation on the dietary behaviour of individuals from Sigatoka Sand Dunes was preceded by a revision of the burial chronology, including 10 AMS radiocarbon dates, and a new analysis of the composition by age of the series to assess the representativeness of the graves and individuals included in the isotopic analyses.

## Archaeological and bioarchaeological contexts of the study of Sigatoka Burial Ground 1

### Sigatoka sand dunes archaeological context and chronology issues

**Fig 1:**
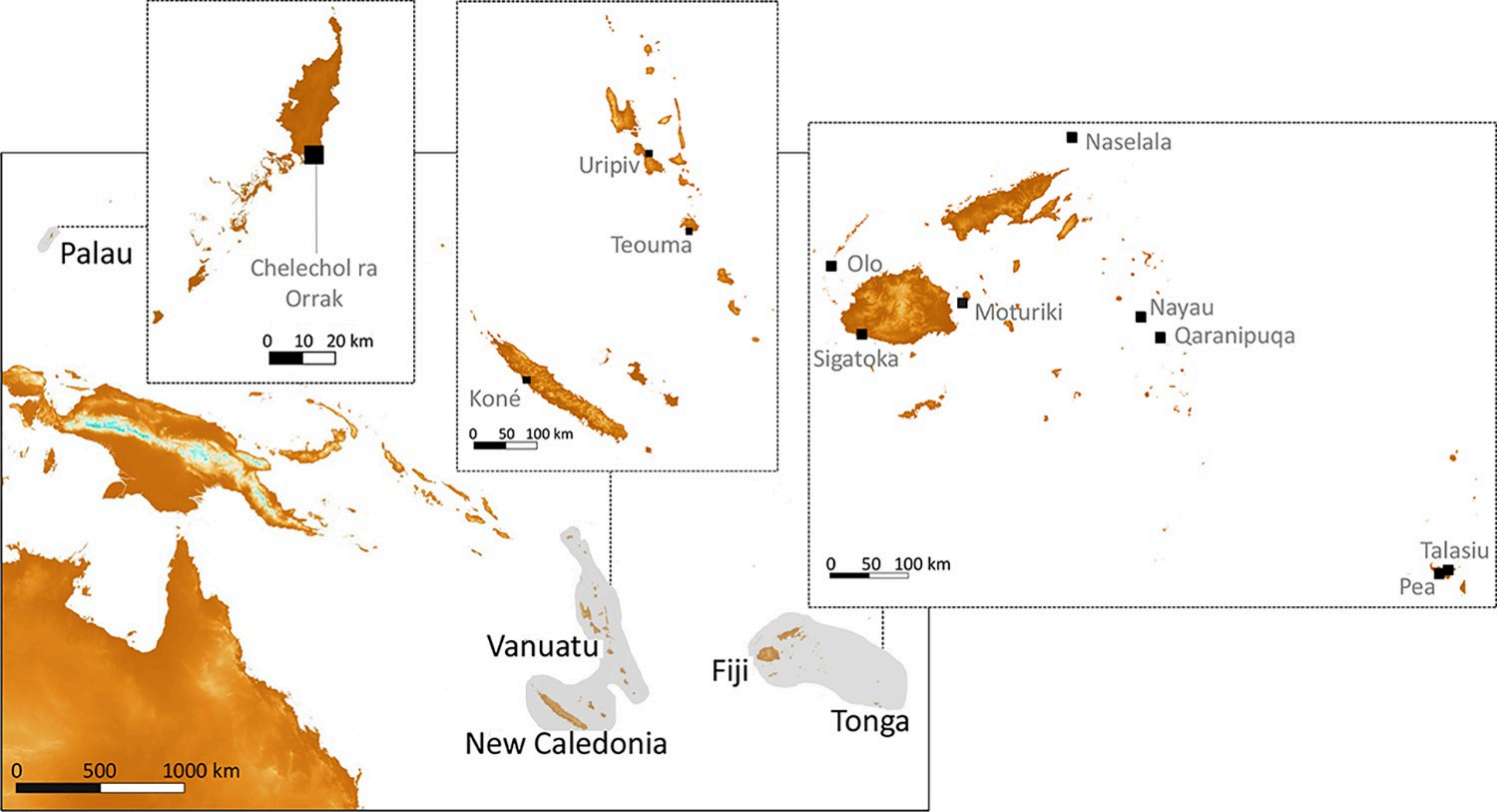
Geographical map showing location of site Sigatoka (Viti Levu, Fiji) and other Southwest Pacific comparisons sites cited in text. The map was created with Shuttle Radar Topographic Mission coordinates available from CIAT–CSI SRTM website [65].

The Sigatoka Sand Dunes archaeological site, located on the southwest coast of Viti Levu, Fiji Islands (Fig 1), is of great importance in defining Fijian and Pacific prehistory [58]. The site (VL16/1) is part of an extensive parabolic dune system, covering an area over 5 km long and 20 to 60 m high, east of the mouth of the Sigatoka River. Since 1944, it has yielded cultural remains of various forms, preservation and nature, ranging from potsherd scatters, fireplace scatters, fire-broken rocks, animal and human bone scatters, to earth oven features, postholes and formal burials protected by cairns [59–62]. Often exposed by erosion, the remains have been recovered in three stratigraphic levels (Levels 1, 2, 3) associated with periods of dune stability [63,64]. These levels represent stages of the whole sequence of the Fijian prehistoric chronology, from the Lapita to the historical periods. Their dating is generally discussed in relation with the discontinuous processes of dune formation and erosion.

Using optical and radiocarbon data, Anderson et al. (2006) [58] showed consistency between radiocarbon and optical ages for Level 1, with dates around 2,800–2,600 cal BP, and Level 3, with dates in the 600–500 cal BP range. They also observed a time lag between the natural formation of Level 2, which starts around 2500–2300 cal BP and human occupations, which extends from 1,700 to 1,300 cal BP [58]. On the other hand, diversity of vessels forms and others characteristics were used to established a four and then five phase ceramic chronology [59,61,66,67]. While some authors have identified the transition from Fiji Plainware to Navatu around 1800 years ago using data from various sites across the Fiji Islands [68–70], this transition appears to occur later at Sigatoka, during the process of formation of Level 2 [59]. Excavations at the easternmost end of the dune revealed two stratigraphically separate but superimposed domestic occupations corresponding to the Fijian Plainware and Navatu phases respectively, with a village occupation dated to 1,433–1,298 cal BP and a subsequent occupation focused on sea salt production dated to1,330–1,266 cal BP [59,71–73]. The change from Fijian Plainware to Navatu is seen as an abrupt cultural modification, interpreted by some as indicating interactions with external population(s) (e.g. [71]].

Human burials have been identified in Levels 2 and 3. Marshall and collaborators [62] have inventoried a total of 119 burials discovered during surveys or archaeological rescue investigations during the 20^th^ century (up to 1998). Their spatial distribution is interpreted as forming three burial grounds, together with several smaller groups of burials or isolated burials scattered across the dune [62: 44–53]. Possible Burial Ground 3, located in the east part of the mapped section of the dune, in Level 3 and above Burial Ground 2, consists of at least 18 interments in deep pits probably related to the last 500 years. Burial Ground 2, which is associated with Level 2, consist of at least 13 poorly preserved burials in an area measuring 25 by 15 m located at about 200 m west of Burial Ground 1. Excavation at Burial Ground 2 did not reveal any coral or basalt fragments covering the burials, some of which consisted of multiple inhumations containing two to four individuals in flexed position following an east–west orientation [52: 110–114,62,74]. Burial Ground 1, where the individuals studied here were found, is the largest burial area. Located in the eastern most part of the dune, it has yielded at least 61 burials associated with Level 2 [62], including 55 (52 labelled and 3 fragmentary) individuals buried in 35 graves excavated by Best [50,57]. A further six interments were subsequently uncovered on the edge of Burial Ground 1 [62: 48–49].

Some scholars have associated the Level 2 burials with the 1,700–1,300 cal BP interval (radiocarbon dates on wood charcoal) [58]. Burley [59,71] raises the possibility that Burial Ground 1 is more closely associated with the Fijian Plainware village occupation found in the easternmost part of the dune and dated to 1,433–1,298 cal BP [31,32,73]. So far, the contemporaneity between the village and Burial Ground 1 has not been established using radiocarbon determinations. Only one burial was radiocarbon-dated with a result outside the chronological range proposed for the village (burial FC1, 1,870±70 BP (Wk–996b), [50]). This result is regarded as questionable due to inadequate bone pre-treatment for radiocarbon dates and unknown diet [75], leaving us with a lack of direct dates for the mortuary contexts.

### Sigatoka Burial Ground 1 graves and individuals

**Fig 2:**
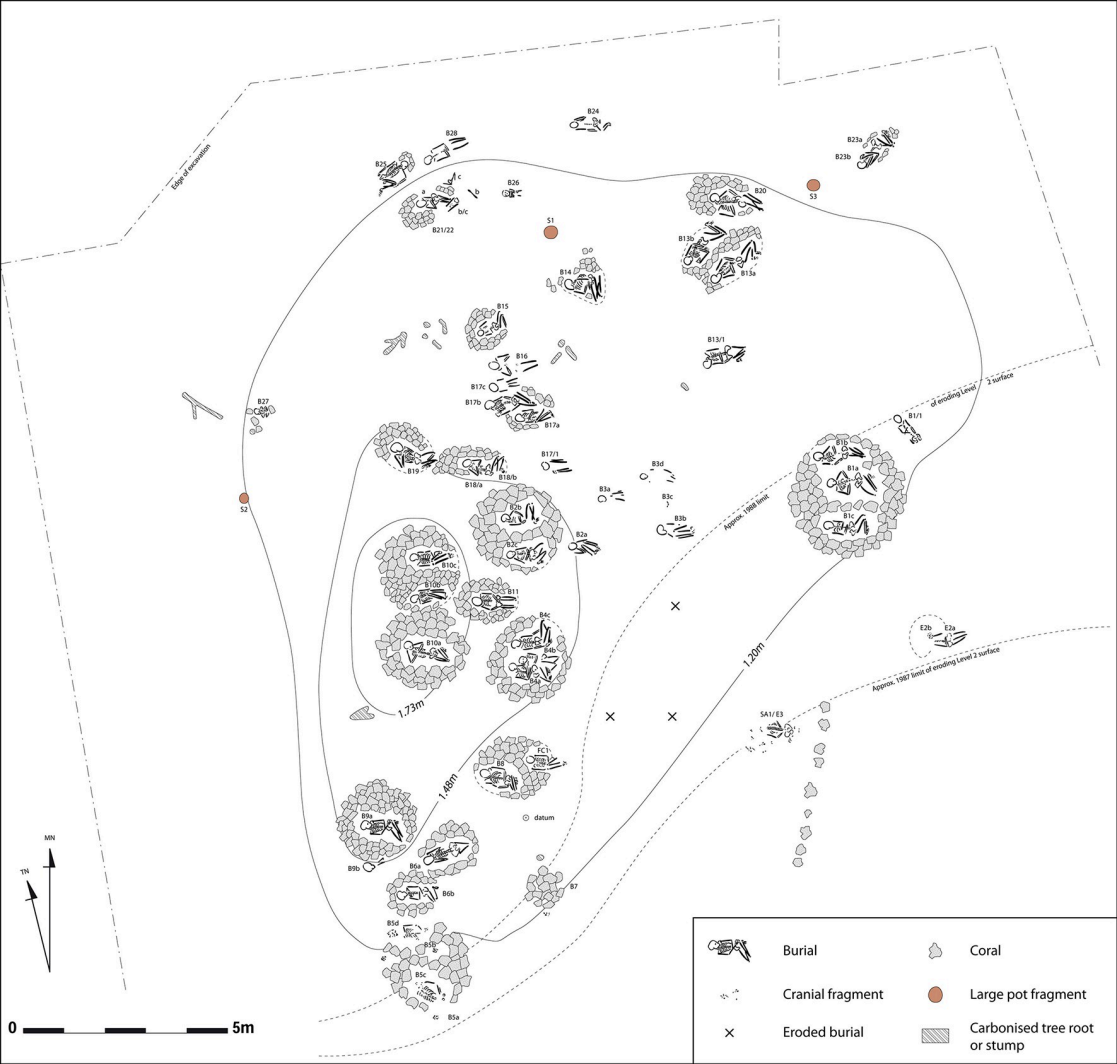
Map of Sigatoka Burial Ground 1. Grave distribution function of topography, burials B10a,b,c are at the highest point (modified from [57] by Hemmamuthé Goudiaby). Reprinted from [57] under a CC BY license, with permission from Simon Best, original copyright 1989.

**Fig 3:**
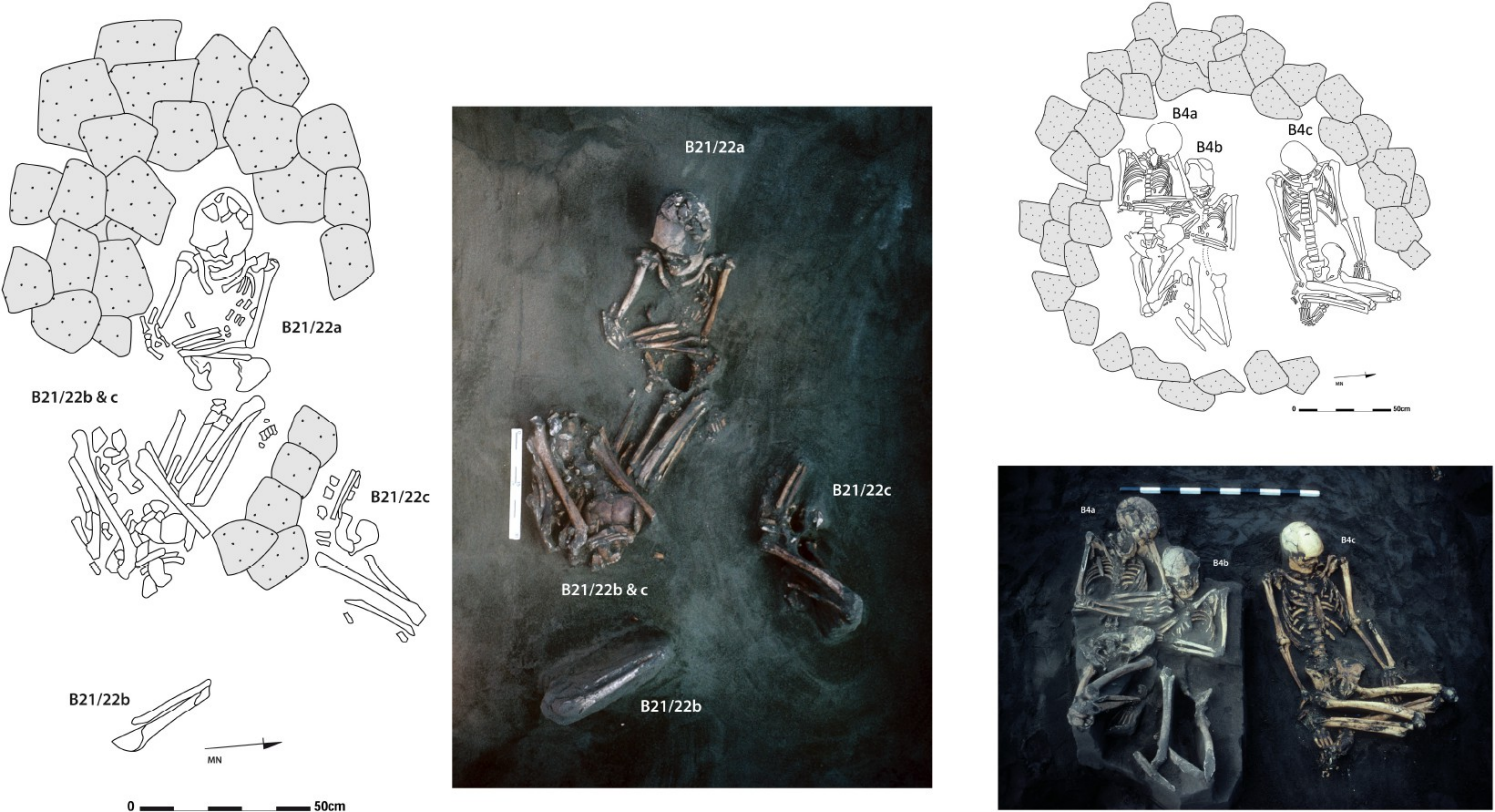
Examples of Sigatoka Burial Ground 1 graves. Left: B21/22 illustrate the single case of successive inhumations at the same location, under cairn. Right: B4 illustrating multiple simultaneous inhumations under cairn (Photos and drawings courtesy Simon Best, modified and assembled by Hemmamuthé Goudiaby). Reprinted from [57] under a CC BY license, with permission from Simon Best, original copyright 1989.

The 55 burials excavated by Best in Burial Ground 1 were in an area measuring 23 by 15 m [57] (Fig 2). They display a consistent pattern of positioning and orientation. The majority of individuals, male, female and children, were buried on the back with the lower limbs flexed to varying degrees, following an east–west orientation. The burial treatment of at least some individuals may have been more complex, comprising a stage of defleshing as indicated by the sharp–force trauma observed on several bones and individuals [75]. Associated artefacts were rare, one stone adze and few shell artefacts, and found in only four burials (B10a, B17, B19, FC1). Most of the graves (n = 20 out of 35) were capped with a cairn of coral or basalt blocks (Figs 2 and 3). These cairn graves contained equal numbers of males and females, and, children in few cases. Graves without a cairn (n = 15) contained mainly females and children (n = 13 out of 15), only one or two were identified as male [28,76]. Some of these cairn graves (n = 8) were single inhumations, while others (n = 12) consisted of group interments of two to four individuals. In one case (B21/22), individuals (two females and an adolescent) were buried successively in the same location (Fig 3), and perhaps in a second case corresponding to the interment of a young child (6–7 years, B9b) found in the cairn of a female (B9a). In the 10 others, individuals were buried as cadavers during a single mortuary event involving several individuals (Fig 3). This practice of burying individuals together at the same time has been interpreted as reflecting a social or familial link, that bound the deceased during their life [57]. This practice can also be seen as an evidence of contemporaneity between the individuals involved.

The spatial organisation of the graves, following the crest of the dune, does not show a specific pattern in the form of strict alignments or rows. Rather the spatial pattern seems to correspond to localised clusters of graves [57] (Fig 2). Their location in relation to the topography of the site appears to be significant. Cairn graves containing males (B10a, b, c), in one case with a number of artefacts (B10a), were found at the highest point of the site, while graves containing females and other individuals were at a lower elevation. Female and child burials, which lacked superstructures and less systematic in their position and orientation, were located toward the northern fringes of the cemetery. This pattern has been interpreted as reflecting social differentiation within Sigatoka community [28: 170–173], with individuals buried under cairns at higher elevations having a higher status in society [57]. It has also been speculated that the spatial separation of Burial Grounds 1 and 2 may reflect the spatial segregation of different segments of the same community [62: 110–111].

Inter-islands contacts may have influenced Sigatoka population lifestyles and dietary practices, with migrants introducing new resources and expertise. Some scholars, using morphological data from skeletal remains from Burial Ground 1 and conducing comparative analysis, suggest that early inter–islands contacts between Viti Levu population and Melanesian region populations already occurred by the beginning of the first millennium CE [80]. As cranial elements exhibit morphological affinities with populations from New Caledonia and Loyalty, in particular, others argue for a later movement of population from North Melanesia [28,77]. Indeed, some levels of population movement between Fiji, Vanuatu, and North Melanesia are recognized during the last millennium CE [78]. Migrants identified through strontium analysis, mainly associated with the Vuda Phase (450–350 year ago) of the Fijian chronology [32] were buried in the Sigatoka Sand Dune. Interestingly, although controversial, the results of an early ancient DNA (mtDNA) study showed that the two individuals tested from Burial Ground 1 display the presence of two copies of the 9 b.p. repeat, a feature common in the mitochondrial genome of present–day populations from the Melanesian region [79].

Assessment of health status points to general good sanitary conditions and adequate nutrient intake during both childhood and adulthood [28,76,80,81]. Low rates of linear enamel hypoplasia (LEH), absence of *cribra orbitalia* were recorded in both children and adults from Burial Ground 1. Signs of infection, seen in three males (B10a, B5c) and female (B18c), and healed trauma, seen in six males (B4c, B10b, B10a, B17b) and females (B4a, B14), are rare. Osteoarthritis and occupational skeletal changes, which occur in both males and females, are also rare, particularly in the postcranial skeleton [76]. Marked joint changes were recorded in the glenoid fossa of some males (n = 3) and females (n = 2). Oral health is good, with the exception of high levels of tooth wear (dentin exposure accounts for about 70% of observations, [76] and *antemortem* tooth loss [28,76,81]. There is limited evidence of dental caries and periodontal disease and an unusual wear patterns in 6 females (B2c, B3b, B3d, B4b, B6a, B9a) and one male (B8), suggesting non–dietary use of teeth for at least some of the individuals [28,80,81]. Sexual differentiation in dental wear, *antemortem* tooth loss and temporo–mandibular joint arthritis patterns of co–variation points to sex specific activities with males chewing kava [80] and females perhaps processing fibrous plant material for fishing nets and lines [81].

## Material & methods

Ethic statement: Sigatoka Sand Dune skeletal remains are stored at the Fiji Museum. Permission for the described analysis was obtained from the Fiji Museum. Collection of samples for exportation and destructive analyses was approved by the Fiji Museum.

### Radiocarbon dates

Thirteen human bone samples from Sigatoka Burial Ground 1 were tested for AMS radiocarbon determinations at the Waikato Radiocarbon Lab (Hamilton, New Zealand) and at the Poznan Radiocarbon Laboratory, Poznan, Poland (S1 Table). All dates were calibrated using OxCal 4.4 software [82] and the SHCal20 calibration curve, with correction for the marine contribution to human diet [83,84]. For the correction, we use the linear extrapolation between terrestrial (−21 ‰) and marine (−12 ‰) *δ*^13^C endpoints to obtain an approximation of the marine contribution to diet, as proposed in Petchey study of early Pacific series [20,85,86]. ΔR and its uncertainty were calculated using the weighted average of the 10 nearest points of the site using the Marine20 database [87].

### Age at death and sex variation analysis

We have focused here on age at death indications provided by subadult mortality variation in order to detect anomalies of representation and structure in the age distribution of the Sigatoka Burial Ground 1 series. For this analysis, 41 adults (over 20 years old) were combined into a single group and 11 subadults were distributed into five age categories [0] year, [1–4] years, [5–9] years, [10–14] years, [15–19] years using data presented in Pietrusewsky and collaborators [76]. Sigatoka Burial Ground 1 mortality tables and curves were reconstructed from the raw data by calculating mortality quotients (aQx) for each age category. These were then compared to our model of natural mortality and to two mortality profiles of series that experienced epidemics and famine. Our non–sex specific model of natural mortality is established using Lederman life tables [88] for pre–vaccination populations with life expectancy at birth between25 to 35 years, for which Lederman provides means values and standards deviation that allow the calculation of quotient confidence interval at 95% (Réseau 100, Tableau 100 MF, p 52) [89,90]. Epidemic and famine profiles were constructed using European series related to the 16^th^ century plague resurgence (Belgium, Dendermonde, archaeological context, [91,92]) and 19^th^–century famine context (Ireland, Workhouse (1841–1851), register, data in [93]). Sex ratio was calculated for individuals over 20 years old using sex estimations provided in Pietrusewsky and collaborators original report [76].

### Isotopic analysis: Sampling strategy, dietary assessment approach

Our sample differs from that of Phaff et al. (2016) [31] in several respects. It includes individuals and paired bone–tooth samples not represented in that publication and for consistency we propose here a new set of analyses performed with the same protocol and analytical measurements, rather than combining Phaff et al.’s data and ours. Our isotopic study uses teeth (all molars, 24 M3, 4 M2 and 2 M1) and bone samples collected on 38 adult individuals among the 41 labelled at Burial Ground 1. These 38 individuals are represented by either a bone or tooth samples, 30 of which represented by paired bone–tooth. The later stage of adult life [94] was documented by bone samples taken preferentially from the skull (n = 13) and long bones (n = 11) as well as the mandible (n = 7) and other skeletal elements (vertebra, scapula and phalanx, n = 7) (S2 Table). The third molar was preferentially selected (n = 24), followed by the second molar for four individuals (B3d, B8a, B10c, B21/22b) and the first molar for two (B10a, B10b). As described in AlQahtani and collaborators’ chart [95], the half distal part of the first molar root captures biological information corresponding to an age of 6.5–9.5 years, the second molar to 10.5–13.5 years and the third molar to the period between 15–20 years. Ten bioarchaeological and mortuary markers: sex, morphology [96], bucco–dental lesions related to physiological stress (LEH, caries, *premortem* tooth loss, and evidence for periodontal disease, data in Pietrusewsky and collaborators [76,81]), and graves characteristics [57] were used to analyse intra–individual isotopic variations. Funerary, biological and health data for each individual are reported in S2 Table. The isotopically studied sample comprises 16 males and 22 females, with 8 individuals are aged below 40 years old and 30 above, based on original data from Pietrusewsky and collaborators [76].

In order to better evaluate the dietary habits and food procurement strategies of the individuals buried at Sigatoka Burial Ground 1, we compared our data with those from other earlier and contemporaneous series from the Southwest Pacific. To this end, we selected data from 112 individuals in 11 publications (Table 1). These include data from coastal localities in Palau [23], Vanuatu [13,17,18,21], New Caledonia [85], Fiji [19,27,85,97,98] and Tonga [24,85]. Table 1 summarises the contextual information and dietary interpretations provided in the original publications and Fig 1 shows their geographical locations. The data selected for our purposes represent individuals older than 5 years with collagen integrity criteria meeting Ambrose’s recommendations [99] (S3 Table). These were divided in three temporal groups using direct dates of the human skeletal remains and/or controlled stratigraphic information available in the original publications (Table 1).

**Table 1:** Chronological and dietary contexts of Southwest Pacific Islands individuals selected for comparison with Sigatoka Burial Ground 1 individuals (individuals and sites with insufficient chronological data [22] have been omitted).

Archipelago	Site	3000–2800 BP (G1) Initial settlement	2800–2500 BP (G2) Later occupation	2500–2000 BP (G3) Later occupation	References
Palau (Babeldaob)	Chelechol ra Orrak		Early inhabitants, marine and terrestrial resources exploitation	Established community, marine and terrestrial diets	[23,100]
Vanuatu (Efate)	Teouma (2–3–A–B)	Colonising group, marine and terrestrial resources exploitation			[5,10,13,78]
Vanuatu (Efate)	Teouma (7C)			Established community, Terrestrial subsistence	[11,13]
Vanuatu (Uripiv)	Uripiv		Established horticulture, Terrestrial and marine reef subsistence	Established horticulture, Terrestrial and marine reef subsistence	[21,101]
New Caledonia (Grande Terre)	Koné		Initial occupation, mixed contribution from both land and marine environments	Established horticulture (taro), marine subsistence	[85,102,103]
Fiji (Yasawa, Waya)	Olo		After colonization, Reliance on nearshore resources including terrestrial foods		[19,104]
Fiji (Moturiki)	Naitabale		Establishment time, with mixed marine–terrestrial diet, dominated by shellfish and lesser amount of terrestrial animals		[97]
Fiji (Lau, Lakeba)	Qaranipuqa			Established community, predominantly terrestrial subsistence	[46,65]
Fiji (Lau, Nayau)	Na Masimasi		Earliest human occupation at site, diet primarily based on plant foods and broad–based diverse inshore subsistence		[23]
Fiji (Cikobia i–Ra)	Naselala			Established community, significant contribution of marine shellfish and/or coral reef fish	[98]
Tonga (Tongatapu)	Talasiu		Established settlement, main reliance on inshore and reef resources		[24,105,106]
Tonga (Tongatapu)	Pea			Established community, transition from marine to terrestrial emphasis	[21,65]

The interpretation of human isotope values aims to identify the dietary resources that were preferentially consumed. The most reliable modelling of the human diet requires knowledge of the isotopic variability of foods available in the local environment of the population under study This baseline is now well–described [24,49,107] thanks to more than 400 stable carbon and nitrogen isotope ratios showing the wide isotopic variability of the local food resources [24]. To infer the consumed dietary resource, we used a theoretical isotope fractionation between food and consumer tissue, of 3 to 6 ‰ for nitrogen, and 0 to 2 ‰ for carbon, at collagen level. We include a +5 ‰ offset due to fractionation during carbon incorporation from fresh food (plant, flesh) into collagen tissue [4,108] and a correction for modern food (+0.86 ‰ for marine ecosystems and +1.5 ‰ for terrestrial ecosystems [13,49]. Based on the Pacific baseline, described elsewhere [24: 312], it is possible to differentiate individuals consuming food of different trophic levels within the foodweb (low trophic level: terrestrial C3 and C4 plants and inshore resources such as shellfish and algae; intermediate trophic level: coral reef fish, non–reef fish and freshwater fish; high trophic level: marine mammals and turtles and terrestrial animals) and individuals consuming terrestrial or marine resources.

### Isotopic analysis: Protocols and statistical analysis

Methods of pre–treatment protocol, isotopic measurements and validation criteria are reported in S1 Text. All archaeological and biological criteria were explored with statistical tests performed with R version 3.6.1 with RStudio [109] with the package Rmisc for descriptive analysis. The non–parametric tests Kruskal Wallis and Mann–Whitney tests are used, when differences are detected, post–hoc comparisons are conducted using the FDR correction [110]. A 0.05 probability (*p* < 0.05) is considered significant. Data are visualized using ©Excel and the package ggplot2 and ggpubr in RStudio.

## Results

### AMS radiocarbon dates

**Fig 4:**
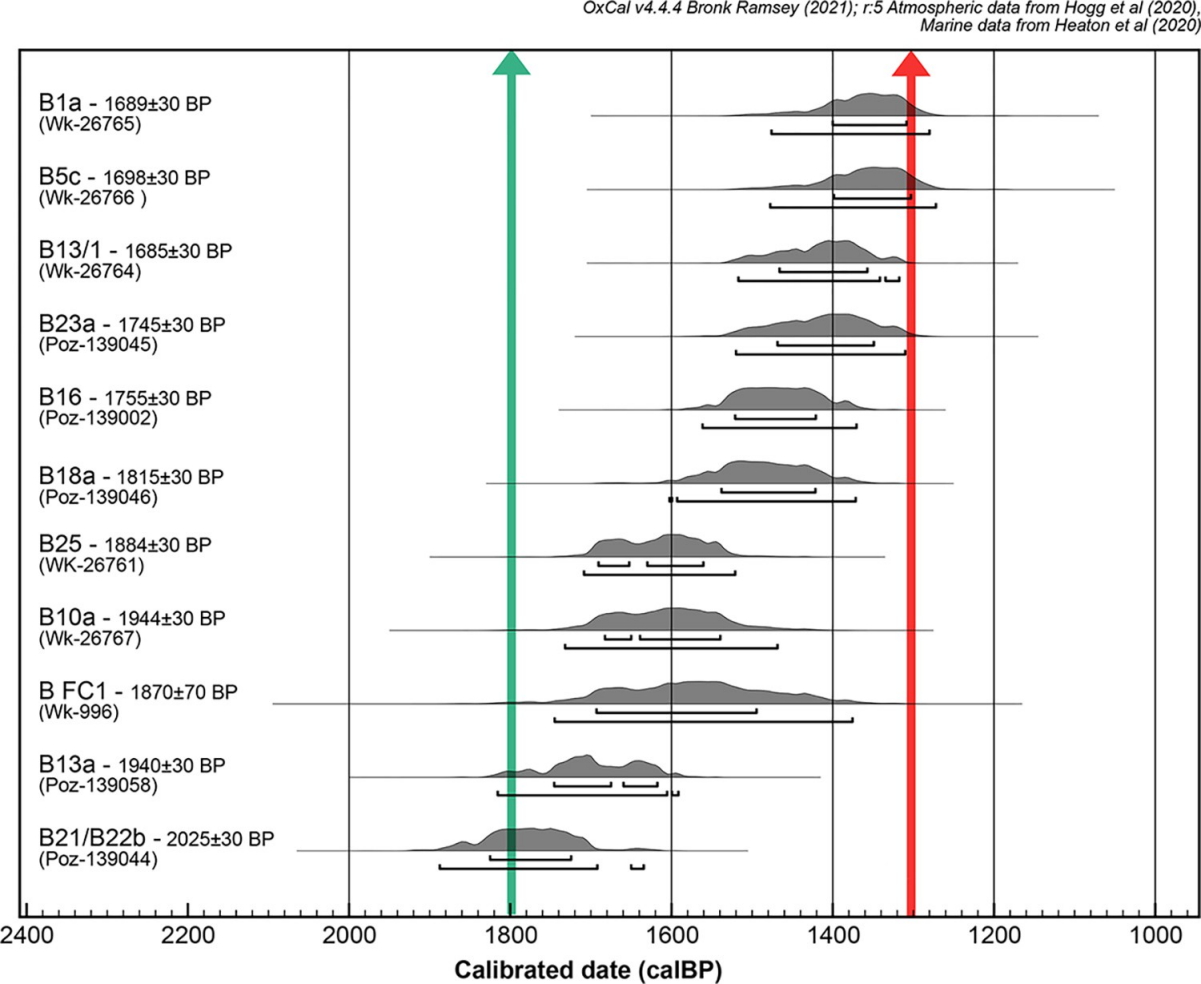
AMS radiocarbon results. Calibrated using using OxCal 4.4 software [82] and SHCal20 calibration curve [83] and hypothesised time of Fijian Plainware/Navatu transition according to Best [57,68] and Clark [70] in green and Burley [73] in red [see Cochrane [67] for a comparison of different ceramic chronologies).

Ten direct AMS radiocarbon dates were obtained from the 13 tested human bone samples from Burial Ground 1 burials. The dates, presented in S1 Table, range from 1,888 to 1,272 cal BP. This range includes the date previously obtained for burial FC1 (1870±70 BP Wk–996, [57]) and overlaps the middle of the first millennium CE (Fig 4). The dates overlap only partially the range of Level 2 radiocarbon dates known to be 1,700–1,300 BP [58] or 1,750–1,550 BP [62: 71], and they are consistent with the range given by optical dating for the start of formation of this level [58]. The individuals buried at Burial Ground 1 represent thus a population that can be considered to be derived from the earliest inhabitants of the area associated with the Sigatoka/Lapita phase [67]. Our results place the burials after the Fijian Plainware Navatu transition as defined by Best and Clark [57,68,70] and before the Navatu phase as defined by Burley and Edinborough (1,330–1,266 cal BP). Some overlap the period associated with the Fijian Plainware village occupation (1,433–1,298 cal BP) [73].

### Age at death and sex composition results

Our analyses of the sex ratio and distribution by age at death tend to support Visser [28] and Pietrusewsky and collaborators [76] views. The ratio of males to female is almost the same (sex ratio = 0,77; χ^2^ test is not significant (χ^2^ = 3.389 p = 0.066). We also found that quotients of children aged between birth and 4 years old are significantly lower than expected while that of individuals aged between 19 and 5 years are consistent with our model of natural mortality (S4 Table). A deficit of young children, and particularly those under 1year of age, is a feature of catastrophic mortality, including epidemics of plague and famine (e.g. [92,93,111]). However, other characteristics of mortality crises do not co-occur in the Sigatoka studied sample, suggesting rather the possibility of a cultural selection of the individuals buried at Burial Ground 1, with those buried representing only part(s) of a larger community. This hypothesis is consistent with Marshall and collaborators suggestion that the spatial separation of Burial Grounds 1 and 2 represents a spatial segregation of different segments of the same community [62: 110–111].

### Isotopic results

#### Quality check and general results

Of the 30 teeth sampled, seven did not provide sufficient collagen samples for measurements and four collagen samples did not meet quality criteria. Of the 38 bone fragments sampled, five did not provide sufficient extraction yield for isotopic measurements and seven collagen samples had criteria of %C, %N and C:N atomic ratios outside the validation ranges for reliable isotopic data. At the end, 25 bones and 19 teeth have well preserved collagen sample for isotopic analysis and paired bone–tooth measurements are available for 15 individuals (S2 Table).

**Fig 5:**
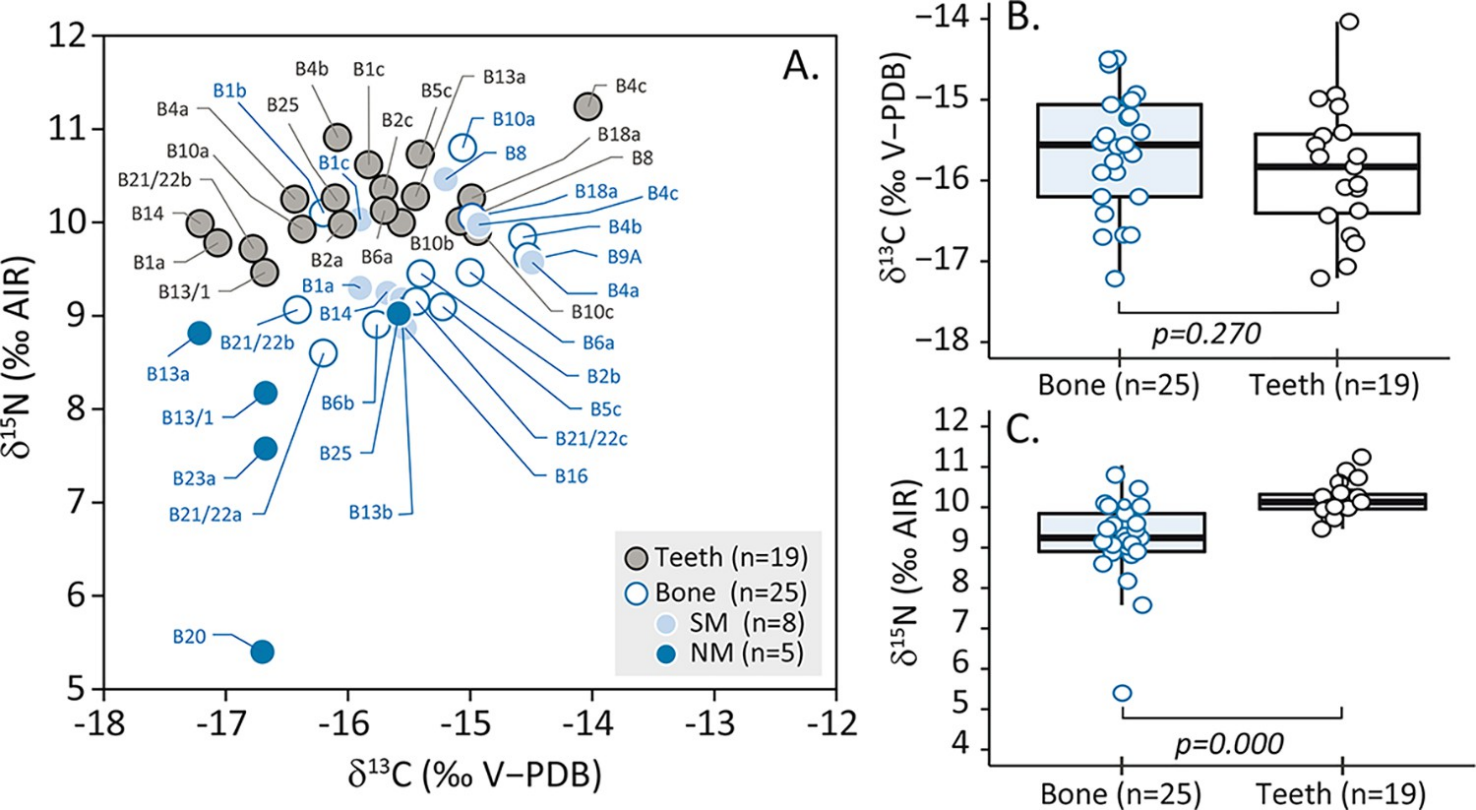
Carbon and nitrogen isotopic ratios of bone and teeth samples for Sigatoka Burial Ground 1 individuals. (Mann-Whitney test between bone values of North Melanesian (NM) and South Melanesian (SM) morphology, p-(*δ*^13^C) = 0.0031, p-(*δ*^15^N) = 0.016 respectively).

Teeth *δ*^13^C values range from −17.2 to −14.0 ‰, and *δ*^15^N values from 9.5 to 11.2 ‰ (mean ± 1SD, −15.9 ± 0.8 ‰; 10.2 ±0.4 ‰, n = 19). Bone *δ*^13^C values range from –17.2 to –14.5 ‰, and *δ*^15^N values range from 5.4 to 10.8 ‰ (−15.6 ±0.7 ‰; 9.2 ± 1.1 ‰, n = 25) (Fig 5). Mann Whitney tests show that teeth *δ*^15^N values are statistically higher than bone values (p = 0.000; Δ^15^N_teeth–bone_ = 0.9‰), whereas differences between teeth and bone *δ*^13^C values are not significant (*p* = 0.270) (Fig 5). For the 15 paired bone–teeth individuals, teeth *δ*^13^C values range from −17.2 to −14.0 ‰, and teeth *δ*^15^N values from 9.5 to 11.3 ‰ (mean±1SD, −15.9 ± 0.9‰; 10.2 ± 0.5 ‰, n = 15) while bone *δ*^13^C values range from –17.2‰ to –14.5‰, and bone *δ*^15^N values from 8.2 to 10.8 ‰ (−15.5 ± 0.8 ‰; 9.5±0.7 ‰, n = 15). Similarly, Mann Whitney tests show that teeth *δ*^15^N values are statistically higher than bone values (*p* = 0.006; Δ^15^N_teeth–bone_ = 0.7‰), while differences between teeth and bone *δ*^13^C values are not significant (*p* = 0.119). Globally, *δ*^13^C and *δ*^15^N bone values present a wide dispersion, 2.7 ‰ and 5.4 ‰, respectively, spanning more than one trophic level. Thus, our results indicate the presence of several dietary behaviours among the Burial Ground 1 individuals.

#### Isotopic values and criteria analysis

**Fig 6:**
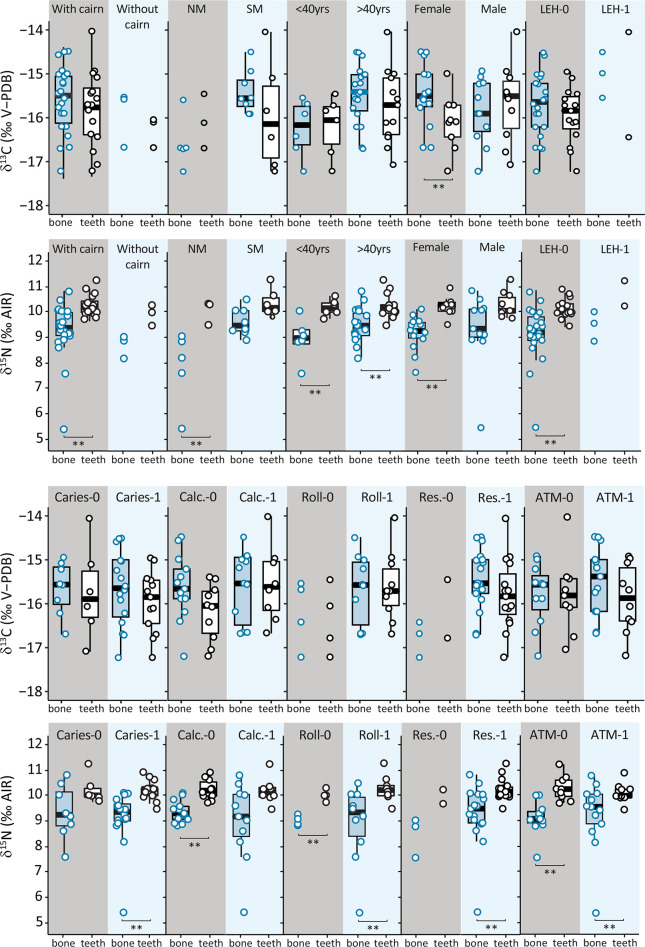
Carbon and nitrogen isotopic ratios of bone and teeth samples for Sigatoka Burial Ground 1 individuals according to archaeological and biological criteria. (See Table 1 for statistics; Without: single individual without cairn; With: several individual under a cairn. For biological criteria: <40: below 40 years old; >40: above 40 years old; NM: North Melanesian; SM: South Melanesian. For health criteria: 0: no lesion; 1: presence of lesion; LEH: Linear enamel hypoplasia; Calc.: calculus; Roll: rolled rim; Res.: alveolar resorption; ATM: premortem tooth loss).

We evaluated the relationship between isotopic values and ten bioarchaeological and mortuary criteria for all available individuals for each type of tissue (regardless their number) (Fig 6, Table 2). Only carbon isotopic ratios show one significant difference, with *δ*^13^C values higher in bone than in tooth for females, suggesting an increased intake of enriched in ^13^C foods, such as marine items, during their life course (Δ^13^C_teeth–bone_ = 0.6 ‰, Mann–Whitney test, *p* = 0.039).

**Table 2:** Sigatoka Burial Ground 1 intra–individual analysis. Descriptive statistics of isotopic data according to tissues (B = bone, T = teeth) and bioarchaeological and archaeological criteria.

		*δ*^13^C (in ‰)				*δ*^15^N (in ‰)				p–value	
	n	min	max	Mean±1SD	Med[IQ1–IQ3]	min	max	Mean±1SD	Med[IQ1–IQ3]	*δ*^13^C	*δ*^15^N
Funerary–Cairn											
B–with	22	–17.2	–14.5	–15.6±0.8	–15.5[–16.1/–15]	5.4	10.8	9.3±1.1	9.4[9.1/10]	0.420	**<0.001**
T–with	16	–17.2	–14.0	–15.8±0.8	–15.7[–16.4/–15.3]	9.7	11.2	10.3±0.4	10.2[10/10.4]		
B–without	3	–16.7	–15.5			8.2	9.0			–––	–––
T–without	3	–16.7	–16.0			9.5	10.3				
Morphology	n										
B–NM	5	–17.2	–15.6		–16.7[–16.7/–16.7]	5.4	9.0		8.2[7.6/8.8]	0.390	**0.036**
T–NM	3	–16.7	–15.4		–16.1[–16.4/–15.8]	9.5	10.3		10.3[9.9/10.3]		
B–SM	8	–15.9	–14.5		–15.5[–15.7/–15.1]	8.9	10.5		9.4[9.2/10]	0.330	0.081
T–SM	6	–17.2	–14.0		–16.1[–16.9/–15.3]	9.8	11.2		10.1[10/10.5]		
Age	n										
B–<40yrs	6	–17.2	–15.5		–16.2[–16.6/–15.7]	7.6	10.0		9[8.8/9.2]	0.940	**0.015**
T–<40yrs	6	–17.2	–15.4		–15.9[–16.6/–15.7]	9.7	10.6		10.1[10/10.3]		
B–>40yrs	19	–16.7	–14.5	–15.4±0.7	–15.4[–15.8/–15]	5.4	10.8	9.3±1.1	9.5[9.1/9.9]	0.300	**0.017**
T–>40yrs	13	–17.1	–14.0	–15.7±0.8	–15.7[–16.4/–15.1]	9.5	11.2	10.2±0.5	10.1[9.9/10.3]		
Sex	n										
B–Female	14	–16.7	–14.5	–15.5±0.7	–15.5[–15.7/–15]	7.6	10.1	9.1±0.7	9.2[8.9/9.5]	**0.039**	**<0.001**
T–Female	9	–17.2	–15.0		–16.1[–16.4/–15.7]	9.5	10.9		10.3[10/10.3]		
B–Male	11	–17.2	–14.9	–15.8±0.7	–15.9[–16.3/–15.2]	5.4	10.8	9.3±1.4	9.3[9/10.1]	0.700	0.150
T–Male	10	–17.1	–14.0	–15.7±0.9	–15.5[–16.2/–15.2]	9.7	11.2	10.2±0.5	10[9.9/10.5]		
LEH	n										
B–0	21	–17.2	–14.5	–15.7±0.7	–15.7[–16.2/–15.2]	5.4	10.8	9.2±1.1	9.2[8.9/9.8]	0.380	**<0.001**
T–0	15	–17.2	–14.9	–15.9±0.6	–15.8[–16.2/–15.5]	9.5	10.9	10.2±0.4	10[10/10.3]		
B–1	3	–15.5	–14.5			8.9	10.0			1.000	0.200
T–1	2	–16.4	–14.0			10.3	11.2				
Caries	n										
B–0	8	–16.7	–14.9		–15.5[–16/–15.2]	7.6	10.8		9.2[8.8/10.1]	0.660	0.230
T–0	6	–17.1	–14.0		–15.9[–16.3/–15.2]	9.8	11.2		10[9.9/10.3]		
B–1	16	–17.2	–14.5	–15.6±0.8	–15.6[–16.3/–15]	5.4	10.1	9.1±1.1	9.3[9/9.7]	0.370	**<0.001**
T–1	13	–17.2	–14.9	–15.9±0.7	–15.8[–16.4/–15.4]	9.5	10.9	10.2±0.4	10.3[10/10.3]		
Calculus	n										
B–0	13	–17.2	–14.5	–15.6±0.7	–15.7[–15.9/–15.2]	8.8	10.1	9.4±0.4	9.3[9.1/9.6]	0.088	**<0.001**
T–0	10	–17.2	–15.4	–16.2±0.6	–16.1[–16.7/–15.7]	9.7	10.9	10.2±0.4	10.2[10/10.5]		
B–1	11	–16.7	–14.5	–15.6±0.8	–15.6[–16.4/–15]	5.4	10.8	9±1.5	9.2[8.4/10]	0.820	0.095
T–1	9	–16.7	–14.0		–15.6[–16.1/–15]	9.5	11.2		10[9.9/10.3]		
Roll	n										
B–0	4	–17.2	–15.5			8.8	9.2			0.970	**0.015**
T–0	4	–17.2	–15.4			9.7	10.3				
B–1	10	–16.7	–14.5	–15.7±0.8	–15.6[–16.5/–15]	5.4	10.5	8.9±1.5	9.3[8.4/9.9]	1.000	**0.029**
T–1	10	–16.7	–14.0	–15.6±0.8	–15.7[–16/–15.2]	9.5	11.2	10.2±0.5	10.2[10/10.3]		
Resorption	n										
B–0	3	–17.2	–16.4			7.6	9.1			–––	–––
T–0	2	–16.8	–15.4			9.7	10.3				
B–1	17	–16.7	–14.5	–15.4±0.7	–15.5[–15.8/–15]	5.4	10.8	9.3±1.2	9.5[8.9/10]	0.140	**0.003**
T–1	15	–17.2	–14.0	–15.8±0.8	–15.8[–16.2/–15.3]	9.5	11.2	10.2±0.4	10.1[10/10.3]		
ATM	n										
B–0	11	–17.2	–14.9	–15.8±0.7	–15.6[–16.2/–15.4]	7.6	10.0	9.1±0.7	9.1[8.9/9.4]	0.760	**<0.001**
T–0	9	–17.1	–14.0		–15.8[–16.1/–15.4]	9.7	11.2		10.3[10/10.6]		
B–1	13	–16.7	–14.5	–15.5±0.8	–15.4[–16.2/–15]	5.4	10.8	9.2±1.4	9.6[8.9/10.1]	0.310	**0.049**
T–1	10	–17.2	–14.9	–15.9±0.8	–15.9[–16.4/–15.2]	9.5	10.9	10.1±0.4	10[10/10.3]		

B: bone; T: Teeth; Mean±1 SD if n≥10; Med = median; Med[IQ1–IQ3] if n≥5.

Without: single individual without cairn; With: several individuals under a cairn. For biological criteria: <40: below 40 years old; >40: above 40 years old; NM: North Melanesian; SM: South Melanesian. For health criteria: 0: no lesion; 1: presence of lesion; LEH: Linear enamel hypoplasia; Calc.: calculus; Roll: rolled rim; Res.: alveolar resorption; ATM: *antemortem* tooth loss.

Nitrogen isotopic ratios show systematically higher values in teeth than in bones (Fig 5). Females have significantly different median nitrogen values, with tooth value being 1.1 ‰ higher than bone value (*p* < 0.001); males do not show such a difference. Individuals of North Melanesian morphology show a weak difference between bone and teeth suggested by a lack of overlap in values. Individuals of South Melanesian morphology show no significant difference. Interestingly, individuals with and without oral lesions show higher isotopic nitrogen values in teeth than in bone. Median nitrogen values are significantly higher in tooth than in bone by +0.8 ‰ for individuals without hypoplasia, +1.1 ‰ for those without calculus, +1.2 ‰ for those without *premortem* tooth loss and by +1.0‰, +0.9 ‰ and +0.4 ‰ for those with caries, rolled rim and *premortem* tooth loss, respectively (Table 2). There is also a significant difference in median nitrogen values of +1.1 ‰ and +0.7 ‰ between bone and teeth for both individuals died below and above 40 years old (*p* = 0.015, *p* = 0.017). Finally, individuals buried under a cairn (grouped or isolated) display significant difference between bone and teeth (Δ^15^N_teeth–bone_ = +0.8 ‰, Mann–Whitney test, *p* < 0.001). Individuals buried without cairn display no difference between tissues.

#### Paired bone–teeth and patterns

**Fig 7:**
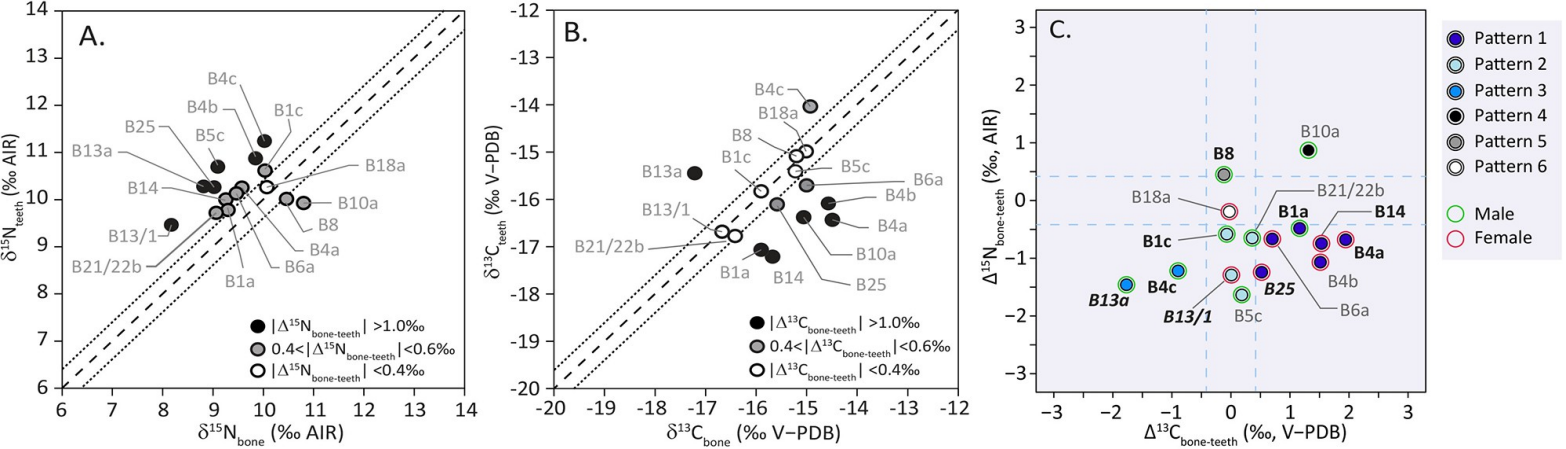
**Paired bone–tooth isotopic values for Sigatoka Burial Ground 1 individuals** (A: Paired bone–tooth nitrogen isotopic ratios. B: Paired bone–tooth carbon isotopic ratios. C: Δ^13^C_bone–teeth_ (*δ*^13^C_bone_–*δ*^13^C_teeth_) and Δ^15^N_bone–teeth_ (*δ*^15^N_bone_–*δ*^15^N_teeth_), a negative Δ value indicate a higher collagen isotopic value in teeth than in bone, corresponding to an impoverishment of heavy isotopes over the life). Labels in Bold: South Melanesian morphology, Labels in Bold–Italic: North Melanesian morphology).

Focusing on the paired bone–teeth isotope values, we found that 13 of 15 individuals, documenting the end of adolescence (between 15–20 years, M3 sampling), have higher tooth nitrogen isotopic ratios than the two others, documenting childhood (B8–M2, B10a–M1) (Fig 7A). We also observe that (i) 14 out of 15 individuals present a difference in nitrogen isotope ratios greater than +0.4 ‰ and (ii) 12 out of 15 show significant ^15^N depletion over the individual’s life course (Δ^15^N_bone–teeth_ < –0.4 ‰) (Fig 7A). Carbon isotopic differences between paired bone–teeth show significant enrichment in ^13^C for seven individuals (B1a, B4a,b, B5c, B6a, B14, B15: Δ^13^C_bone–teeth_ > +0.4 ‰) and depletion of ^13^C for two individuals (B4c, B13a: Δ^13^C_bone–teeth_ < –0.4‰) (Fig 7B). Nine show a significant difference for both carbon and nitrogen isotope values. The isotopic shifts are different for each element (carbon or nitrogen) and do not affect the direction of the shift between paired bone–teeth in the same way (Fig 7C). Thus, the isotopic differences between the two tissues display six different isotopic patterns that can be interpreted as representing different dietary or physiological modifications during life (Fig 7C):

- Pattern 1 corresponds to an increase (> 0.4‰) of *δ*^13^C values and a decrease of *δ*^15^N values from teeth (childhood) to bone (adult). It is the most recurrent pattern, affecting six of the 15 individuals (B25, B6a, B14, B1a, B4a,b). The opposite isotopic changes of the two elements (Δ^15^N_bone–teeth_ < –0.4 ‰, Δ^13^C_bone–teeth_ > +0.4 ‰) suggest an increasing dependence on low trophic level marine resources such as shellfish and seaweed and/or a decrease of animal protein intake from adolescence to adulthood.
- Pattern 2 shows a decrease of *δ*^15^N values of four individuals (B21/22b, B13/1, B5c, B1c). The negative Δ^15^N_bone–teeth_ values (< –0.4‰) indicate a greater reliance on terrestrial plant resources over the course of their life.
- Pattern 3 is characterised by a significant decrease in both carbon and nitrogen isotopic ratios from teeth (childhood) to bone (adult), observed in two individuals (B13a, B4c). With negative values for both Δ^15^N_bone–teeth_ and Δ^13^C_bone–teeth_, these two individuals would have had a lower terrestrial and marine animal protein intake in adulthood than in adolescence.
- Pattern 4 is characterised by an increase in both carbon and nitrogen isotopic ratios visible in only one individual (B10a), suggesting a greater terrestrial/marine animal protein intake in adulthood than in childhood.
- Pattern 5 only shows an increase of *δ*^15^N values in one individual (B8). Although the pattern is different, a similar dietary interpretation to Pattern 4 can be proposed, with a greater reliance on animal proteins in adulthood than in childhood.
- Pattern 6 shows no isotopic change for either carbon and nitrogen, and involves only one individual (B18a), which would support the hypothesis of a similar diet throughout her life.

We also note that nine of the 15 individuals with paired bone–tooth (B1a, B4a,b,c, B6a, B10a, B13a, B14, B25) with significant isotopic shift during life, for both *δ*^15^N and *δ*^13^C, are dispersed across the cemetery (Fig 2: 1/4 NE: B13a; SE: B1a; SW: B6a; NW: B14, B25; SW: B4a,b,c, B10a). Similarly, these individuals do not show any specific dietary trends/patterns in relation with their grave form or mortuary treatment. Of the six individuals with Pattern 1, four were buried with others at the same time under the same cairn, one was interred alone under a cairn and another without a cairn. Contemporaneous individuals (buried simultaneously soon after death) display different patterns like for grave B4 or grave B1. For example, in grave B4 (Figs 3 and 7C), the two females (B4a,b) show a decrease in trophic level associated with an increase in marine food over their lifetime (Pattern 1) whereas the male (B4c, Pattern 3) is characterised by a shift towards a more terrestrial plant protein intake.

Finally, we observe that the five females of Pattern 1 (B4a,b, B6a, B14, B25) and the female of Pattern 2 (B13/1) show a decrease in high trophic level foods from tooth to bone whereas the seventh (B18a, Pattern 6) shows no dietary shift (Fig 7). Six of the eight males (B1a,c, B5c, B21/22b, B4c, B13a) present a similar trend to females, with a decreasing trophic level of food over the life course and two of them (B4c, B13a) also show a marine dietary shift. The last two (B8 and B10a) display a higher consumption of ^15^N enriched food over the life course (positive Δ^15^N_bone–teeth_ values > +0.4‰). Their condition illustrates a shift occurred during the earlier periods of growth (sampling on M1, M2) while the others males and females represent a later period of growth (sampling on M3).

#### Chronological and geographical comparisons

**Fig 8:**
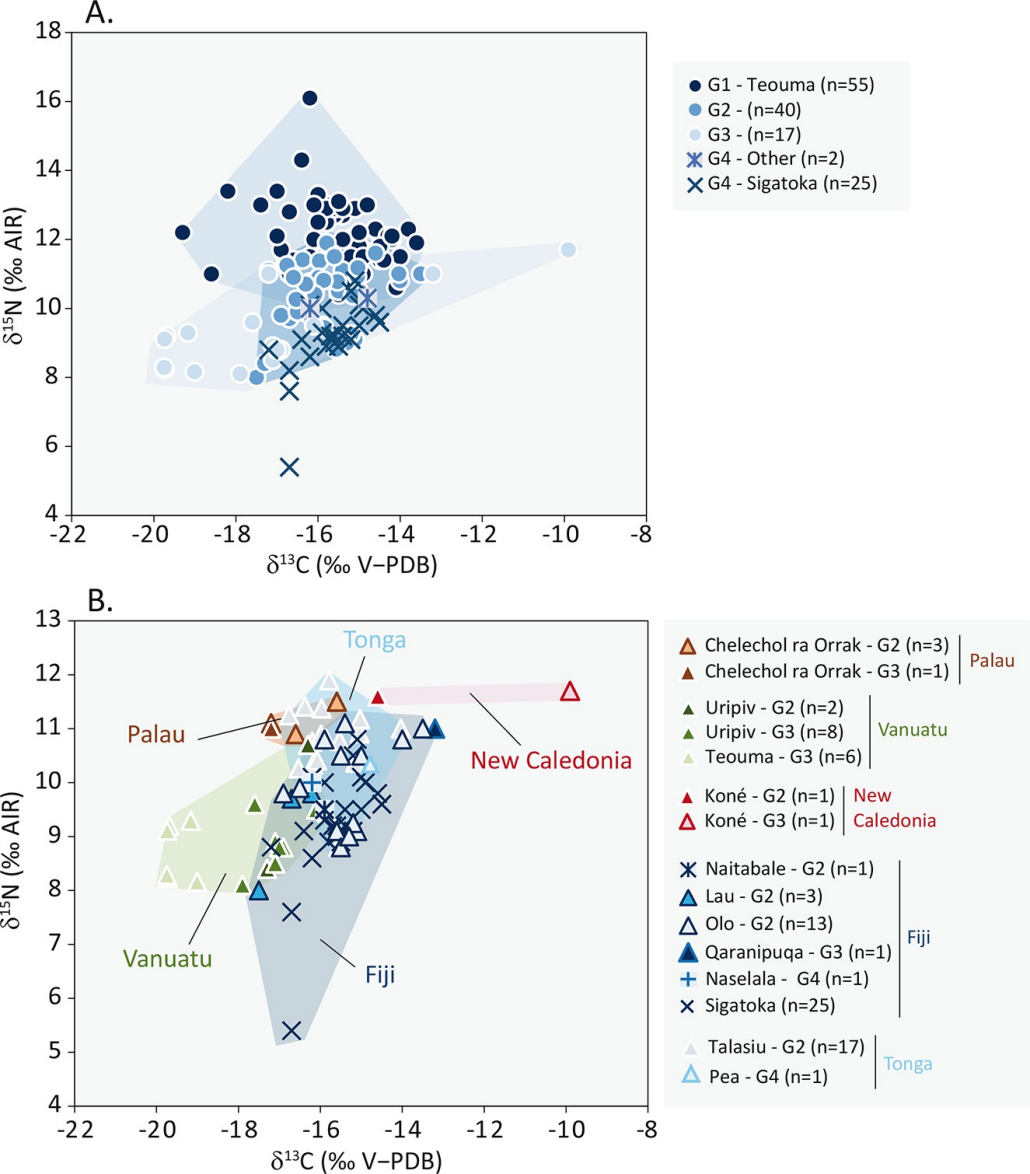
Comparison between Sigatoka Burial Ground 1 individuals *δ*^13^C and *δ*^15^N values.

Comparison of bone collagen isotopic ratios of individuals from Sigatoka Burial Ground 1 and other individuals from Southwest Pacific highlights several trends that may be a function of chronology and geography (Table 3, Fig 8, S3 Table). Sigatoka individuals *δ*^13^C median value is similar to that of the more ancient individuals considered in this study (with respective mean ± 1σ −15.7 ± 1.2 ‰, n–G1 = 55 and −15.8 ± 0.9 ‰, n–G2 = 40) but is significantly higher than that of the more recent individuals (+2.3 ‰ to G3, *p* = 0.001) (Fig 8A). In contrast, Sigatoka individuals *δ*^15^N median value is significantly lower than that of the more ancient individuals studied (−2.9 ‰ to G1 and −1.0 ‰ to G2, respectively 12.1 ± 1.0 ‰, n–G1 = 55 and 10.3 ± 1.0 ‰, n–G2 = 40, with for both groups *p* < 0.001) and is similar to that of the more recent individuals considered in this study.

**Table 3:** Descriptive statistics of isotopic data (bone collagen sample) of Southwest Pacific series dated between 3,000 and 1,400 BP. (Isotopic data were selected in published data considering preservation criteria, age above 5 years, see Table 1 for references and see S3 Table for individual data).

		*δ*^13^C (‰)			*δ*^15^N (‰)			Dietary trends	Mann–Whitney Test–Sigatoka with …	
	n	min	max	Mean±1σ	min	max	Mean±1σ		p–value(*δ*^13^C)	p–value(*δ*^15^N)
**G1–3000–2800 BP**	**55**	**–19.3**	**–13.6**	**–15.7±1.2**	**10.4**	**16.1**	**12.1±1.0**		0.835	**<0.001**
Teouma–Vanuatu	55	–19.3	–13.6	–15.7±1.2	10.4	16.1	12.1±1	Marine~Terrestrial		
**G2–2800–2500 BP**	**40**	**–17.5**	**–13.5**	**–15.8±0.9**	**8.0**	**11.9**	**10.3±1.0**	Marine~Terrestrial	0.822	**<0.001**
**G2 –Fiji**	**17**	**–17.5**	**–13.5**	**–15.6±1**	**8.0**	**11.1**	**9.8±0.9**	Marine~terrestrial & Low ^15^N intake	0.820	0.050
Lau–Fiji	3	–17.5	–16.2	–16.8±0.7	8.0	9.8	9.2±1	Marine~terrestrial & Low ^15^N intake	–––	–––
Olo–Fiji	13	–16.9	–13.5	–15.3±0.9	8.8	11.1	10±0.8	Marine~Terrestrial & Low ^15^N intake	1.000	0.063
Naitabale–Fiji	1	–15.9			9.5			Marine>>Terrestrial	–––	–––
Talasiu–Tonga	17	–16.8	–14.0	–15.8±0.7	8.8	11.9	10.7±0.7	Marine~Terrestrial	0.820	**<0.001**
Uripiv–Vanuatu	2	–17.3	–16.3	–16.8±0.7	8.4	10.7	9.5±1.6	Marine~terrestrial^+^	–––	–––
Chelechol ra Orrak–Palau	3	–17.2	–15.6	–16.5±0.8	10.9	11.5	11.2±0.3	Marine~Terrestrial	–––	–––
Koné–New Caledonia	1	–14.6			11.6			Marine~Terrestrial	–––	–––
**G3–2500–2000 BP**	**17**	**–19.7**	**–9.9**	**–17.3±2.5**	**8.1**	**11.7**	**9.2±1.1**	Marine<<Terrestrial	**0.001**	0.311
**G3 –Vanuatu**	**14**	**–19.8**	**–16.1**	**–18.1±1.3**	**8.1**	**9.6**	**8.8±0.5**	Terrestrial & Low ^15^N intake	**<0.001**	**0.036**
Teouma (7C)–Vanuatu	6	–19.7	–19.0	–19.5±0.3	8.2	9.3	8.7±0.5	Terrestrial & Low ^15^N intake	**0.003**	0.103
Uripiv–Vanuatu	8	–17.9	–16.1	–17.1±0.5	8.1	9.6	8.9±0.5	Terrestrial & Low ^15^N intake	**<0.001**	0.081
Qaranipuqa–Fiji	1	–13.2			11.0			Marine>>Terrestrial	–––	–––
Koné–New Caledonia	1	–9.9			11.7			Marine>>Terrestrial	–––	–––
Chelechol ra Orrak–Palau	1	–17.2			11.0			Marine~terrestrial	–––	–––
**G4–2000–1400 BP**	**27**	**–17.2**	**–14.5**	**–15.6±0.7**	**5.4**	**10.8**	**9.3±1.1**		**–––**	**–––**
Sigatoka–Fiji	25	–17.2	–14.5	–15.6±0.7	5.4	10.8	9.2±1.1	Marine~terrestrial & Low ^15^N intake	–––	–––
Naselala–Fiji	1	–16.2			10.0			Marine~Terrestrial & Low ^15^N intake	–––	–––
Pea–Tonga	1	–14.8			10.3			Marine~Terrestrial	–––	–––

Comparisons between archipelagos show that (i) Sigatoka individuals *δ*^13^C and *δ*^15^N median values are significantly higher than those of the most recent individuals of Vanuatu included in the study (G3–Vanuatu n = 15: +2.2 ‰ in ^13^C, −17.8 ±1.8 ‰, *p* < 0.001 and +0.2 ‰ in ^15^N, 9.0 ± 0.7‰, *p* = 0.036), (ii) only the Sigatoka individuals *δ*^15^N median value is significantly lower than that of the earlier Tongan individuals studied (−1.5 ‰, 10.7 ± 0.7 ‰, *p* < 0.001, n = 17) and (iii) there is no differential isotopic pattern between the Sigatoka and the early Fijian individuals included in the study (G2–Fiji) (Table 3, Fig 8B).

Comparisons between Fijian sites are shown for series of more than 5 individuals. Sigatoka median values for both elements are similar to those at Olo (2,800–2,500 years ago, n = 13). Sigatoka individuals *δ*^15^N median value is significantly lower than that of the more ancient studied individuals from Teouma (−2.9 ‰, 12.1 ± 1.0 ‰, *p* < 0.001, n = 55). Individuals from Sigatoka Burial Ground 1 have a significantly higher *δ*^13^C median value than the more recent individuals of both Teouma 7C and Uripiv sites (respectively +3.8 ‰ −19.5 ± 0.3‰, *p* = 0.003, n = 6 and +1.4 ‰; −17.1 ± 0.5 ‰, *p* = 0.003, n = 8) (Table 3, Fig 8B).

## Discussion

### Chronology

The individuals from Sigatoka Burial Ground 1 constitutes a skeletal series unique in the region for the first millennium CE, with the exception of the two isolated burials from Naselala in North Fiji [98) and Pea in Tonga [85,112] and an unpublished series of graves uncovered also in a dune system in the Bourail region in New Caledonia [113–115]. Our ^14^C dates indicate that the interments took mainly place in the first half of the first millennium CE, with an age range of 1,888 to 1,272 cal BP. Such an age range places 70% of the burials before the Fijian Plainware period (1,433–1,298 cal BP) as defined by Burley and Edinborough [73] (Fig 4). Burials and Fijian Plainware village occupations do not appear to be fully contemporaneous, in contrast with what was previously recognized [31,32]. This temporal difference may account for the inconsistency between the limited abundance of remains of terrestrial fauna and coastal remains recorded for the village locus and the isotopic reconstruction of human diet produced by Phaff and collaborators [31] rather than any other explanation. The range of variation in mortuary practices observed at Burial Ground 1 is probably not related to the transition from Fijian Plainware to Navatu, regardless of the date of that transition (1,800 BP or 1,300 BP) [67] (Fig 4). Our dates indicate that the funerary events were not concentrated in a short period, but rather occurred over a longer period of about 600 years, albeit with no discernible spatial pattern, with older graves scattered throughout Burial Ground 1. It can be noted, however, that the older individuals (B21/22b, B10a, Fig 3) are either a secondary deposit associated with burial B21/22a and a burial (B10a) located at the highest point of the site, that is perhaps foundational of Burial Ground 1 [57] (Fig 2). With 34% of the funerary events involving contemporary deaths of multiple individuals distributed over a long period of time combined with a cultural selection of the interred individuals, the examined skeletal series reflect a discontinuous representation of the population who once inhabited the Sigatoka Sand Dune area.

### Isotopic changes during the lifetime

Higher *δ*^15^N values in teeth (n = 19) than in bone (n = 25) suggest either a diet enriched in ^15^N during dental tissue formation or a depletion of ^15^N in the adult diet (Figs 5 and 6). Given the isotopic variability of food resources in the Southwest Pacific and the trophic enrichment between food and consumer tissues, the observed dietary change over the life course could be related to a differential quantitative contribution of both terrestrial and marine protein and both high and low trophic level resources. However, considering the potential for body protein recycling in the context of physiological stress, such as starvation [53,54], this increase in dental tissue may also indicate a period of stress during childhood.

Dietary changes over the life span have been discussed in terms of physiological stress for other Southwest Pacific individuals, such as in Southeast Solomon, at the Namu site (c. 440–150 cal BP) [45,46], in American Samoa (1,200–100 cal BP) [47] and in Fiji, at the Bourewa site (740–150 cal BP) [48]. It is interesting to note that regardless the broad geographical and chronological contexts covered by these archaeological sites, teeth appear to be regularly enriched in ^15^N compared to bone. Such a broad distribution and inter–site effect is also observed at the intra–site level at Burial Ground 1. Isotopic values were obtained from individuals whose dates of death are widely distributed over time, spanning some 600 years of cemetery use. It is thus difficult to conceive that such a repetitive pattern is consistent with a single physiological process associated with a "starvation effect" for all individuals, a similar process that would have occurred over such a long period of time and with sufficient impact to be recorded in the growing tissues of children and adolescents. The hypothesis of a physiological stress remains complicated to consider as a robust explanation for the systematic elevated *δ*^15^N values in dental tissue compared to bone tissue, because bone tissues are more sensitive to environmental changes than dental tissues [116], though physiological stress, pathology and metabolic syndrome could also have affected the body nitrogen balance of certain individuals [117]. This would require future investigation using modern tooth samples of individuals whose dietary and health information can be monitored. Moreover, as the age of death of individuals, before or after 40 years, is not associated with higher teeth *δ*^15^N values compared to bones, other criteria must be considered to explain the isotopic trend observed over the life course.

A dietary change in protein trophic level over the life course is apparent in 14 of the 15 Sigatoka Burial Ground 1 individuals represented by paired bone–tooth data. Only one individual (B18a, Pattern 6) would have maintained a similar diet throughout life (Fig 7C). The dental isotopic signal documents the period between 15–20 years for 12 of the individuals (M3, n = 12) while the dental i signal of the three others refers to the periods between 6.5–9.5 years (M1, B10a) and 10.5 to 13.5 years (M2, B8a, B21/22b), respectively. These results suggest that the dietary change generally occurred in a short time, between the end of adolescence and adulthood. It could finally support another interpretation of the intra–individual isotopic changes for the Sigatoka Burial Ground 1 individuals. Firstly, these individuals display a pattern suggesting good sanitary condition [71]. Pietrusewsky and collaborators [81] do not report any pathological lesions that could be related to metabolic disorder and our mortality profile, in agreement with the palaeopathological evidence, show no evidence of famine. Second, the observation that the nitrogen isotopic ratios are systematically higher in M1 and M2 dentine than in adult bone (whatever the archaeological, social or health criteria) does not support a physiological stress induced by a poor health status during dental tissue formation (Table 2, Fig 6). The pattern is more consistent with the hypothesis of dietary changes than with health disruption and physiological stress, a hypothesis proposed by other authors for other Southwest Pacific series [45,46,48]. Interestingly, B10a individual, an old male who has suffered from extreme dental *antemortem* loss and moderate dental wear during his adulthood, displays no evidence of dietary stress that could have occurred during infancy. He is the only individual to present an isotopic pattern consistent with a dietary shift towards more marine fish ingestion at the end of his life. Perhaps explaining their originality, their grave, very close to the grave of two other males at the highest point and amongst the earliest of the site (Fig 2), is considered to be the foundation of Burial Ground 1 [57].

Our results do not show clear connection between isotopic changes and the location of the graves in the cemetery, nor with the burial treatment. The exception is grave B4, which contains three contemporaneous bodies buried together under the same cairn (Fig 3). B4a and B4b, two females, experienced a similar dietary change during their life, with a decrease in trophic level associated with an increase of marine food (Pattern 1). The third individual, a male (B4c, Pattern 3), is characterised by a shift towards a more terrestrial plant protein intake (Fig 7). Their association at death may reflect a similar life history as suggested by Best [57], with the two females having similar dietary behaviours at different stages of their life. Interestingly, these two females share the most common dietary pattern (Pattern 1), which shows a change towards more shellfish and algae intake over the life course. Our results clearly emphasise sexual variation, which was expected on the basis of ethnographic information [118–120], but not discernible in the analysis of the adult bone alone [31]. They are therefore consistent with the hypothesis of a specific use of coastal and reef resources by female adolescents and women, as already put forward by isotopic data measured on other Fijian series [16,121]. For now, if a higher marine resource intake has existed at Sigatoka, it would have distinguished mainly the female diet over life course. Could this be an illustration of a social difference? If so, contrary to what was reported previously [31,32], this would not be reflected in the funerary practices, but rather in the daily life activities. Dental markers analysed for individuals from Burial Ground 1 show sex–specific trends in wear, *antemortem* tooth loss and temporo–mandibular arthritis [80,81]. Our isotopic results suggest that this male/female social distinction existed at Sigatoka since the childhood, whereas Fijian ethnohistoric sources mainly inform on lifeways of male of high rank [16,119,120].

### Temporal and geographical variations across Southwest Pacific

Focusing on the bone isotope data of individuals from Sigatoka Burial Ground 1, one individual (B20) has the lowest human *δ*^15^N value of all the Southwest Pacific data used in this study (5.4 ‰). Their health condition and layout of their grave, which is similar to that of other individuals found at the site, do not allow us to propose any hypothesis other than the influence of a particular diet to explain this super–low value. The elemental carbon and nitrogen composition of this bone sample confirms a well–preserved collagen, and to our knowledge, no physiological stress that induces a negative ^15^N balance has been described in the literature. The only alternative to identify which ^15^N–depleted foods could have been consumed by this individual would be to reinforce the local isotopic baseline. Other individuals (B13/1, B13a, B23a, B25), with a North Melanesian morphology and buried in the north part of Burial Ground 1, also present low *δ*^15^N values (5.4 to 9.0 ‰) (Fig 5). Their *δ*^13^C and *δ*^15^N values are significantly lower than those of individuals with a South Melanesian morphology dispersed in other parts of the cemetery (8.9 to 10.5 ‰) (*p* = 0.0031, *p* = 0.016 respectively) (Fig 2), a particularity also noted for their mortuary and health characteristics [28,57,59]. Interestingly, these individuals (B13/1, B13a, B23a, B25) present similar *δ*^15^N values as post-Lapita individuals from Teouma 7C and Uripiv (8.1 to 9.6 ‰) (Figs 5 and 8, Table 3).

Based on the Southwest Pacific isotopic baseline and the diet–consumer expected trophic level enrichment (see 3.3 section), the differences in *δ*^13^C and *δ*^15^N values between individuals from Burial Ground 1 and individuals dated to 3,000 to 2,000 years ago suggest (i) a decrease in the consumption of foods from trophic levels (lower *δ*^15^N values) over time (Fig 8A, Table 3) and (ii) the persistence of a marine protein intake similar to that observed for individuals dated to 3,000 to 2,500 years ago. The dietary pattern described would be consistent with a mixed marine and terrestrial diet as defined for Lapita and Late Lapita individuals [17,24,107] but (i) with consumption of very low trophic level foods (shellfish, seaweed, algae), and (ii) without the contribution of terrestrial C3 plants observed in the individuals dated to 2,500 to 2,000 years ago [18]. In other words, our data suggest that individuals from Sigatoka Burial Ground 1, whose radiocarbon age range from 1888 to 1272 cal BP, have not yet undergone a dietary transition towards the consumption of horticultural products, except for a few individuals (B13/1, B13a, B23a, B20, B25) characterized by more vegetarian diets. Indeed, diachronic dietary changes appear to be uneven across the Southwest Pacific region. A different timing of acquisition of the horticultural component in diet has already been suggested for the case of Tongatapu island, Kingdom of Tonga, with a date at the beginning of the second millennium CE [25].

The site–to–site comparisons indicate that individuals from Sigatoka Burial Ground 1 have higher nitrogen isotopic ratios than individuals dating to 2,500–2,000 years ago in Vanuatu (Uripiv and Teouma 7C) and are similar to the early Fijian individuals from Waya (Olo) island. Inter–site comparisons also show an overlap in isotopic values between the Sigatoka individuals and (i) the 2,800–2,000 years ago individuals from Moturiki (Naitabale), Lau (Nayau), and Lakeba (Qaranipuqa), all from Fiji, and (ii) the two contemporaneous individuals from Naselala in Northern Fiji and Pea in Southern Tonga. By showing that the Sigatoka population is closer to other Fijian populations than to populations from other archipelagos, these results suggest a pattern of dietary consumption of marine resources linked to geography, which in turn may be linked to environmental and/or social factors. It has already been observed that island characteristics, including size, climate, and soil, are important factors that can shape subsistence strategies at a local scale [19]. The example of individuals from Bourewa (south coast of Viti Levu), dated to around 740–150 cal BP, shows a strong dependence on marine resources [48], though the adoption of horticultural practices is widely attested at that time in other Fijian island and locations. The influence of a social and cultural factor remains a plausible explanation for the diversity of dietary practices observed in the Pacific over time.

On the other hand, based on terrestrial sedimentary and soils records of biomass burning, Roos and collaborators [122] suggest an anthropogenic transformation of the landscape around Sigatoka and the use of swidden practices around 1,600–1,800 cal BP [122], a hypothesis that apparently contradicts with our interpretation of isotopic data from Sigatoka Burial Ground 1. A horticultural/agricultural production, perhaps not yet efficient enough to provide a sufficient amount of food to sustain the inhabitants of the region could explain why marine and coastal resources remained largely consumed by the majority of the individuals until the middle of the first millennium CE. This situation contrasts with that at the Teouma7C and Uripiv sites, where the consumption of horticultural products was adopted from about 2,500 years ago [18,21]. The changes in dietary behaviour observed at Teouma between 3,000 and 2,500 years ago could be related, at least in part, to the arrival of new comers whose presence in the archaeological record has been shown through morphometric and genetic analyses [123–126]. Conversely, the trend observed at Sigatoka, which clearly suggests more stable dietary habits over a longer period of time, could be associated with a close intergenerational transmission of cultural practices. Cultural evidence tends to confirm continuity, with no change in ceramic data and in Sigatoka Level 2 paleosol before 1,350 years ago [71].

Whether the lack of diachronic dietary change demonstrated by individuals from Sigatoka Burial Ground 1 is actually due to sociocultural or environmental factors, or a combination of both, remains an open question. Sociocultural factors may have been played a key role as our analysis of the composition per age of the series suggests a sociocultural selection of the individuals buried at Burial Ground 1 from a larger group. A lack of pressure on marine coastal is another possibility, as suggested for the Talasiu individuals (Tongatapu, Tonga) [24]. The continued consumption of marine resources implies either that the size of the living group was in line with the resources available or that the group may have been able to effectively manage their marine resources in a sustainable manner. Settlement contraction or demographic decline during the first millennium CE has been suggested for several small islands of Western and Eastern Fiji [68,127–129]. Such a reduction of population number may have created a positive retroactive balance between population need and available food resources. This is supported by the good health status and the lack of physiological stress in individuals from Burial Ground 1 [81] and our isotopic results.

## Conclusion

Phaff and collaborators [31] have emphasised a social distinction among individuals buried at the Sigatoka Sand Dunes site. Some individuals with sophisticated grave architecture would have had a preferential access to animal proteins, as opposed to individuals buried in simpler graves [31]. For the authors, the limited abundance of terrestrial fauna and the absence of coastal resources observed at the village site would not be consistent with their food reconstruction, hence their hypothesis of food resource exchange between Sigatoka inhabitants and other nearby sites [31]. Our direct ^14^C dates on skeletal remains from Burial Ground 1 (1,888 to 1,272 cal BP) demonstrate the absence of full contemporaneity between the village occupation and the dead, challenging this inter–or intra island exchange hypothesis. Our dates show that the funerary events occurred over a period of time of about 600 years with no discernible spatial pattern. Our results indicate that dietary changes over the life span are multiple. Overall, *δ*^15^N values are higher in tooth than in bone while no *δ*^13^C difference was observed between the two tissues. In contrast to previous studies suggesting that the enrichment in ^15^N in dental tissue would be related to physiological stress [45,46,48], our analysis does not support a link with particular health conditions for the Sigatoka individuals studied. Rather, their good health suggests a positive relationship between the population and its (geographical, cultural or social) environment, a diet of sufficient quantity and quality to ensure the survival of the group, and even a possible food self–sufficiency (as suggested by our isotopic data), not requiring mandatory potential interaction with other groups. Our analysis of paired bone–teeth analysis reveals six different isotopic patterns, suggesting different dietary changes throughout life, from the childhood/adolescence to adulthood, shaped by a differential contribution of marine resources associated with the consumption of foods of different trophic levels. These specific dietary patterns do not seem to be related to the funerary treatment given to the individuals, but rather to their way of life. In fact, our results indicate a sex–specific change in diet during life. Females had acquired a greater contribution of marine food resources with age, especially coastal and inshore items. This result is consistent with the hypothesis of a specific use of coastal and reef resources by women, as already emphasised on the basis of osteological data [28,80,81], suggesting a sexual division of labour and food acquisition in the Sigatoka community.

Our regional comparisons highlight the specificity of the diet of individuals from Sigatoka Burial Ground 1 compared to other earlier and contemporaneous Southwest Pacific series. They appear to have more dietary similarities with other Fijian individuals than with individuals from other archipelagos. The individuals from Sigatoka Burial Ground 1 have consumed more coastal resources, such as shellfish, algae and seaweed than the early individuals from Teouma in Vanuatu, Talasiu in Tonga and Chelechol ra Orrak in Palau. They have also consumed more marine resources than the Vanuatu individuals (Uripiv, Teouma 7C) dating to 2,500–2,000 years ago, who were more focused on a terrestrial vegetarian diet. What might be the common factor at the Fijian Islands level to explain this stability of the dietary practices over time, in contrast to the individuals from archipelagos further west? External interactions and arrivals of new comers during the first millennium of island occupation, at least in Vanuatu, make a clear difference between western and eastern parts of the South West Pacific region, while geological, ecological and environmental features clearly distinguish the island of Viti Levu Island, where the Sigatoka Sand Dune site is located, from the other Fijian islands with more limited agricultural potential.

## Supporting information

S1 Table Excel data table with individuals submitted to AMS radiocarbon dates and results. Calibrated using using OxCal 4.4 software (Bronk Ramsey 2009 [82]) and SHCal20 calibration curve (Hogg et al. 2020 [83]), marine data from (Heaton et al 2020 [84]).
(XLSX)

S2 Table Excel data table with biological, archaeological data and elemental and isotopic measurements for individuals from Sigatoka Burial Ground 1 (the skeletal remains are currently stored at Fiji Museum, Suva, Fiji). (XLSX)

S3 Table Excel data table with isotopic data of individuals selected for comparisons between Sigatoka Burial Ground 1 and other Southwest Pacific series. Individuals are distributed into 4 groups, as described in Table 1 for G1, G2 and G3. The fourth group (G4) dated to c. 2000–1400 years ago comprises the individuals of Sigatoka Burial Ground 1 and two other contemporaneous individuals from Fiji (98) and Tonga (85) (S3 Table).
(XLSX)

S4 Table Mortality profile of Sigatoka Burial Ground 1 individuals compared to the Ledermann life tables (88) (life expectancy at birth 25–35 years old) and to mortality profiles associated with plague and famine. (XLSX)

S1 Text Supporting information for the stable isotope analysis (PDF). (DOCX)

## References

[1] Katzenberg A. Stable isotope analysis: A tool for studying past diet, demography, and life history. In: Katzenberg A, Saunders SR, editors. Biological anthropology of the human skeleton. Hoboken, New Jersey: John Wiley & Sons, Inc; 2008. p. 413–41.

[2] Schoeninger MJ. Diet Reconstruction and Ecology Using Stable Isotope Ratios. In: Larsen CS, editor. A Companion to Biological Anthropology. Oxford, UK: Wiley-Blackwell; 2010. p. 445–64.

[3] Schoeninger MJ, DeNiro MJ. Nitrogen and carbon isotopic composition of bone collagen from marine and terrestrial animals. Geochimica et Cosmochimica Acta. 1984;48:625–39.

[4] Bocherens H, Drucker D. Trophic level isotopic enrichment of carbon and nitrogen in bone collagen: case studies from recent and ancient terrestrial ecosystems. International Journal of Osteoarchaeology. 2003;13:46–53.

[5] Kirch P. On the road of the winds: An archaeological history of the Pacific Islands before European contact. Second edition. Berkeley: University of California Press; 2017.

[6] Leclerc M, Flexner J. Archaeologies of Island Melanesia: current approaches to landscapes, exchange and practice. First edition, Canberra Australia: ANU Press; 2019.

[7] Florin SA, Fairbairn AS, Nango M, Djandjomerr D, Hua Q, Marwick B, et al. 65,000 years of changing plant food and landscape use at Madjedbebe, Mirarr country, northern Australia. Quaternary Science Reviews. 2022;284:107498.

[8] Denham T. Early Agriculture and Plant Domestication in New Guinea and Island Southeast Asia. Current Anthropology. 2011;52(S4 (The Origins of Agriculture: New Data, New Ideas)):S379–95.

[9] Clark G, Petchey F, Hawkins S, Reepmeyer C, Smith I, Masse WB. Distribution and extirpation of pigs in Pacific islands: a case study from Palau. Archaeology in Oceania. 2013;48(3):141–53.

[10] Roberts P, Douka K, Tromp M, Bedford S, Hawkins S, Bouffandeau L, et al. Fossils, fish and tropical forests: prehistoric human adaptations on the island frontiers of Oceania. Philos Trans R Soc Lond B Biol Sci. 2022;377:20200495. doi: 10.1098/rstb.2020.0495 35249390 PMC8899615

[11] Roberts P, Hixon S, Hamilton R, Lucas M, Ilgner J, Marzo S, et al. Assessing Pleistocene-Holocene climatic and environmental change in insular Near Oceania using stable isotope analysis of archaeological fauna. Journal of Quaternary Science. 2023;38(8):1267–78.

[12] Ambrose SH, Butler BM, Hanson DB, Hunter-Anderson RL, Krueger HW. Stable isotopic analysis of human diet in the Marianas archipelago, Western Pacific. American Journal of Physical Anthropology. 1997;104:343–61. doi: 10.1002/(SICI)1096-8644(199711)104:3&lt;343::AID-AJPA5&gt;3.0.CO;2-W 9408540

[13] Kinaston RL, Buckley HR, Valentin F, Bedford S, Spriggs M, Hawkins S, et al. Lapita diet, subsistence strategies and methods of animal husbandry in Remote Oceania: new stable isotope evidence from the 3000-year-old Teouma site, Efate Island, Vanuatu. PLoS ONE. 2014;9:e90376. 24598939 10.1371/journal.pone.0090376 PMC3944017

[14] Leach BF, Quinn CJ, Lyon GL, Haystead A, Myers DB. Evidence of Prehistoric Lapita diet at Watom Island, Papua New Guinea using stable isotopes. New Zealand Journal of Archaeology. 2000;20:149–59.

[15] Petchey F, Green RC. Use of three isotopes to calibrate human bone radiocarbon determinations from Kainapirina (SAC), Watom Island, Papua New Guinea. Radiocarbon. 2005;47(2):181–92.

[16] Valentin F, Bocherens H, Gratuze B, Sand C. Dietary patterns during the late prehistoric/historic period in Cikobia island (Fiji): insights from stable isotopes and dental pathologies. Journal of Archaeological Science. 2006;33:1396–410.

[17] Valentin F, Buckley HR, Herrscher E, Kinaston RL, Bedford M, Spriggs M, et al. Lapita subsistence strategies and food consumption patterns in the community of Teouma (Efate,Vanuatu). Journal of Archaeological Science. 2010;37:1820–9.

[18] Valentin F, Herrscher E, Bedford S, Spriggs M, Buckley HR. Evidence for social and cultural change in central Vanuatu between 3000 and 2000 BP: comparing funerary and dietary patterns of the first and later generations at Teouma, Efate. The Journal of Island and Coastal Archaeology. 2014;9:381–99.

[19] Field JS, Cochrane EE, Greenlee DM. Dietary change in Fijian prehistory: isotopic analyses of human and animal skeletal material. Journal of Archaeological Science. 2009;36(7):1547–56.

[20] Petchey F, Spriggs M, Bedford S, Valentin F. The chronology of occupation at Teouma, Vanuatu: Use of a modified chronometric hygiene protocol and Bayesian modeling to evaluate midden remains. Journal of Archaeological Science: Reports. 2015;4:95–105.

[21] Kinaston R, Bedford S, Richards M, Hawkins S, Gray A, Jaouen K, et al. Diet and Human Mobility from the Lapita to the Early Historic Period on Uripiv Island, Northeast Malakula, Vanuatu. PLoS ONE. 2014;9:e104071. doi: 10.1371/journal.pone.0104071 25140807 PMC4139273

[22] Kinaston RL, Anson D, Petchey P, Walter R, Robb K, Buckley H. Lapita diet and subsistence strategies on Watom Island, Papua New Guinea: New stable isotope evidence from humans and animals., American Journal of Physical Anthropology. 2015;157:30–41. doi: 10.1002/ajpa.22685 25641394

[23] Stone JH, Fitzpatrick SM, Krigbaum J. Stable Isotope Analysis of Human Diet at Chelechol ra Orrak, Palau. Bioarchaeology International. 2019;3(2):142–56.

[24] Herrscher E, Fenner JN, Valentin F, Clark G, Reepmeyer C, Bouffandeau L, et al. Multi-isotopic analysis of first Polynesian diet (Talasiu, Tongatapu, Kingdom of Tonga). Journal of Archaeological Science: Reports. 2018;18:308–17.

[25] Fenner JN, Herrscher E, Valentin F, Clark G. An Isotopic Analysis of Late Lapita and State Period Diets in Tonga. Archaeological and Anthropological Sciences. 2021;13(1):22.

[26] Allen MS, Craig JA. Dynamics of Polynesian subsistence: Insights from archaeofauna and stable isotopes studies, Aitutaki, Southern Cook Islands. Pacific Sciences. 2009;63(4):477–506.

[27] Jones S, Quinn RL. Prehistoric Fijian diet and subsistence: integration of faunal, ethnographic, and stable isotopic evidence from the Lau Island Group. Journal of Archaeological Science. 2009;36:2742–54.

[28] Visser EP. The people of Sigatoka. PhD. thesis, University of Otago; 1994.

[29] Leach F, Quinn C, Morrison J, Lyon G. The use of multiple isotope signatures in reconstructing prehistoric human diet from archaeologial bone. New Zealand Journal of Archaeology. 2003; 23:31–98.

[30] Phaff BN. Human dietary and mobility patterns of a prehistoric population from Sigatoka, Fiji: a reconstruction using stable isotope analysis [Master Thesis]. [Vancouver]: Department of Anthropology, University of British Columbia; 2012.

[31] Phaff B, Burley D, Richards MP. Dietary isotope patterns and their social implications in a prehistoric human population from Sigatoka, Fiji. Journal of Archaeological Science Reports. 2016;5:680–8.

[32] Cheung C, Burley DV, Phaff B, Richards MP. A palaeomobility study of the multi-period site of Sigatoka, Fiji, using strontium isotope analysis. Journal of Archaeological Science: Reports. 2018;17:762–74.

[33] Ericson JE. Strontium isotope characterization in the study of prehistoric human ecology. Journal of Human Evolution. 1985;14(5):503–14.

[34] Richards MP, Mays S, Fuller BT. Stable carbon and nitrogen isotope values of bone and teeth reflect weaning age at the Medieval Wharram Percy site, Yorkshire, UK. American Journal of Physical Anthropology. 2002;119(3):205–10. doi: 10.1002/ajpa.10124 12365032

[35] Fuller BT, Richards MP, Mays SA. Stable carbon and nitrogen isotope variations in tooth dentine serial sections from Wharram Percy. Journal of Archaeological Science. 2003;30:1673–84.

[36] Herrscher E. Alimentation d’une population historique. Analyse des données isotopiques de la nécropole Saint-Laurent de Grenoble (XIIIe-XVe siècle, France). Bulletins et Mémoires de la Société d’Anthropologie de Paris. 2003;15(3–4):149–269.

[37] Kaupová S, Herrscher E, Velemínský P, Cabut S, Poláček L, Brůžek J. Urban and rural infant feeding practices and health in Early Medieval Central Europe (9th–10th century, Czech Republic). American Journal of Physical Anthropology. 2014;155(4):635–51. doi: 10.1002/ajpa.22620 25256815

[38] Beaumont J, Gledhill A, Lee-Thorp J, Montgomery J. Childhood diet: A closer examination of the evidence from dental tissues using stable isotope analysis of incremental human dentine. Archaeometry. 2013;55(2):277–95.

[39] Fernández-Crespo T, Czermak A, Lee-Thorp JA, Schulting RJ. Infant and childhood diet at the passage tomb of Alto de la Huesera (north-central Iberia) from bone collagen and sequential dentine isotope composition. International Journal of Osteoarchaeology. 2018;(28):542–51.

[40] Mion L, Herrscher E, Blondiaux J, Binet E, Andre G. Comportements alimentaires en Gaule du Nord: étude isotopique du site de l’Îlot de la Boucherie (IIIe–Ve siècles apr. J.-C.) à Amiens. Bulletins et Mémoires de la Société d’Anthropologie de Paris. 2016;28(3):155–75.

[41] Miller MJ, Dong Y, Pechenkina K, Fan W, Halcrow SE. Raising girls and boys in early China: Stable isotope data reveal sex differences in weaning and childhood diets during the Eastern Zhou era. American Journal of Physical Anthropology. 2020;(172):567–85. 10.1002/ajpa.24033 PMC7496748 32141612

[42] King CL, Buckley HR, Petchey P, Kinaston R, Millard A, Zech J, et al. A multi-isotope, multi-tissue study of colonial origins and diet in New Zealand. American Journal of Physical Anthropology. 2020;172(4):605–20. doi: 10.1002/ajpa.24077 32424829

[43] Cheung C, Fernandez-Crespo T, Leïa M, Di Giusto M, Goude G, Macdonald R, et al. Micro-punches vs. Micro-slices for Serial Sampling of Human Dentine: Striking a Balance between Improved Temporal Resolution and Measuring Additional Isotope Systems. Rapid Communications in Mass Spectrometry. 2022;36:e9380. 35986908 10.1002/rcm.9380 PMC9787592

[44] Kramer RT, King CL, Buckley HR, Jaouen K, Boyd DA, Kiko L, et al. Strontium (87Sr/86Sr) isotope analysis of the Namu skeletal assemblage: A study of past human migration on Taumako, a Polynesian Outlier in the eastern Solomon Islands. American Journal of Physical Anthropology. 2021;174(3):479–99. doi: 10.1002/ajpa.24179 33305833

[45] Kinaston RL, Buckley HR. Isotopic insights into diet and health at the site of Namu, Taumako Island, Southeast Solomon Islands. Archaeological and Anthropological Sciences. 2017;(9):1405–20.

[46] Stantis C, Buckley HR, Commendador AS, Dudgeon JV. Expanding on incremental dentin methodology to investigate childhood and infant feeding practices on Taumako (southeast Solomon Islands). Journal of Archaeological Science. 2021;126(3):105294.

[47] Eerkens JW, Bartelink EJ, Bartel J, Johnson PR. Isotopic insights into dietary life history, social status, and food sharing in American Samoa. American Antiquity. 2019;84(2):336–52.

[48] Stantis C, Buckley HR, Kinaston RL, Nunn PD, Jaouen K, Richards MP. Isotopic evidence of human mobility and diet in a prehistoric/protohistoric Fijian coastal environment (c. 750–150 BP). American Journal of Physical Anthropology. 2016;159:478–95. doi: 10.1002/ajpa.22884 26487418

[49] Kinaston RL, Buckley HR. The stable isotope analysis of Prehistoric human diet in the Pacific islands with an emphasis on Lapita. In: Summerhayes G, Buckley HR, editors. Pacific Archaeology: Documenting the past 50,000 years. Dunedin: University of Otago studies in Archaeology; 2013. p. 91–107.

[50] Best S. A preliminary report on the Sigatoka burials. Domodomo. 1987;3:2–15.

[51] Katzenberg MA, Lovell NC. Stable isotope variation in pathological bone. International Journal of Osteoarchaeology. 1999;9(5):316–24.

[52] Fuller B, Fuller J, Sage N, Harris D, O’Connell T, Hedges R. Nitrogen balance and *δ*^15^N: why you’re not what you eat during pregnancy. Rapid Communications in Mass Spectrometry. 2004;18:2889–96. 15517531 10.1002/rcm.1708

[53] Fuller BT, Fuller BT, Sage NE, Harris DA, O’Connell TC, Hedges R. Nitrogen balance and 15N: why you’re not what you eat during nutritional stress. Rapid Communications in Mass Spectrometry. 2005;19:2497–506. 16106342 10.1002/rcm.2090

[54] Mekota AM, Grupe G, Ufer S, Cuntz U. Serial analysis of stable nitrogen and carbon isotopes in hair: monitoring starvation and recovery phases of patients suffering from anorexia nervosa. Rapid Communications in Mass Spectrometry. 2006;20(10):1604–10. doi: 10.1002/rcm.2477 16628564

[55] Rysava K, McGill RAR, Matthiopoulos J, Hopcraft JGC. Re-constructing nutritional history of Serengeti wildebeest from stable isotopes in tail hair: seasonal starvation patterns in an obligate grazer. Rapid Communications in Mass Spectrometry. 2016;30(13):1461–8. doi: 10.1002/rcm.7572 27321833 PMC5089620

[56] Hillson SW. Dental anthropology. Cambridge: Cambridge University Press; 1996. 373 p.

[57] Best S. The Sigatoka dune burials (Site VL 16/1). Suva: Fiji Museum; 1989. (Report on file at Fiji Museum).

[58] Anderson A, Roberts R, Dickinson W, Clark G, Burley D, de Biran A, et al. Times of sand: sedimentary history and archaeology at the Sigatoka Dunes, Fiji. Geoarchaeology. 2006;21(2):131–54.

[59] Burley DV. Mid-sequence archaeology at the Sigatoka sand dunes with interpretive implications for Fijian and Oceanic culture history. Asian Perspectives. 2005;44(2):330–48.

[60] Gifford EW. Archaeological Excavations in Fiji. University of California Press. Berkeley and Los Angeles; 1951. (Anthropological Records; vol. 3).

[61] Birks L. Archaeological excavations at Sigatoka dune site, Fiji. Bulletin of the Fiji Museum. 1973;1:1–68.

[62] Marshall Y, Crosby A, Matararaba S, Wood S. Sigatoka: The Shifting Sands of Fijian Prehistory. University of Southampton. Southampton: Oxbow; 2000. (Monograph).

[63] Dickinson WR, Burley DV, Nunn PD, Anderson A, Hope G, de Biran A, et al. Geomorphic and archaeological landscapes of the Sigatoka Dune site, Viti Levu, Fiji: interdisciplinary investigations. Asian Perspectives. 1998;31(1):1–31.

[64] de Biran A. The Holocene geomorphic evolution of the Sigatoka Delta, Viti Levu, Fiji Islands. PhD thesis, University of the South Pacific; 2001.

[65] Jarvis A, Reuter HI, Nelson A, Guevara E. Hole-filled SRTM: version 4: data grid. International Centre for Tropical Agriculture (CIAT). 2008. Web publication/site, CGIAR Consortium for Spatial Information. http://srtm.csi.cgiar.org/

[66] Green RC. A suggested revision of the Fiji sequence. Journal of the Polynesian Society. 1963;72:235–53.

[67] Cochrane E. Ancient Fiji: Melting pot of the Southwest Pacific. In: Cochrane E, Hunt TL, editors. The Oxford Handbook of Prehistoric Oceania. Oxford:Oxford University Press; 2014. p. 206–230.

[68] Best S. Lakeba: The Prehistory of a Fijian Island. PhD Thesis, Anthropology Department, University of Auckland; 1984.

[69] Best S. Lapita: A view from the east. Auckland: New Zealand Archaeological Association; 2002. (Monograph 24).

[70] Clark GR. Post-Lapita Fiji: cultural transformation in the mid-sequence. The Australian National University; 1999.

[71] Burley DV. Fijian polygenesis and the Melanesian/Polynesian divide. Current Anthropology. 2013;54(4):436–62.

[72] Burley DV, Taché K, Purser M, Naucabalavu J. An archaeology of salt production in Fiji. Antiquity. 2011;85:1187–200.

[73] Burley DV, Edinborough K. Discontinuity in the Fijian archaeological record supported by a Bayesian radiocarbon model. Radiocarbon. 2014;56(1):295–303.

[74] Crosby A. Further Burials at the Sigatoka Sand Dunes (Site VL 16/1). Suva: Fiji Museum; 1991. (Report to the Fiji Museum).

[75] Jones S, Walsh-Haney H, Quinn R. Kana tamata or feasts of men: an interdisciplinary approach for identifying cannibalism in Prehistoric Fiji. International of Journal of Osteoarchaeology. 2015;25(2):127–45.

[76] Pietrusewsky M, Douglas M, Ikehara-Quebral R. The Human Osteology of the Sigatoka Dune Burials (Site VL 16/1), Viti Levu, Fiji Islands. Honolulu: Department of Anthropology, University of Hawai’i; 1994.

[77] Visser EP, Green MK. Prehistoric Oceanic biological variation: Sigatoka, Lapita, and Polynesia. In: Galipaud JC LI J. C., editor. The Pacific from 5000 to 2000 BP: Colonisation and Transformations. Paris: IRD; 1999. p. 161–87.

[78] Bedford S, Spriggs M. The Archaeology of Vanuatu: 3,000 Years of History across Islands of Ash and Coral. Oxford University Press; 2014. (The Oxford Handbook of Prehistoric Oceania).

[79] Hagelberg E, Clegg JB. Genetic polymorphisms in prehistoric Pacific islanders determined by analysis of ancient bone DNA. Proc Biol Sci. 1993;252(1334):163–70. doi: 10.1098/rspb.1993.0061 8391704

[80] Visser EP. Skeletal evidence of kava use in prehistoric Fiji. Journal of the Polynesian Society. 1994;103(3):299–317.

[81] Pietrusewsky M, Toomay Douglas M, Ikehara-Quebral RM. Skeletal and dental health: The bioarchaeology of the human skeletons from the Sigatoka Sand Dunes Site, VL 16/1, Viti Levu, Fiji. Journal of Pacific Archaeology. 2017;8(2):63–78.

[82] Bronk Ramsey C. Bayesian analysis of radiocarbon dates. Radiocarbon. 2009;51:337–60.

[83] Hogg A, Heaton T, Hua Q, Palmer J, Turney C, Southon J, et al. SHCal20 Southern Hemisphere calibration, 0–55,000 years cal BP. Radiocarbon. 2020;62(4):759–78.

[84] Heaton T, Köhler P, Butzin M, Bard E, Reimer R, Austin W, et al. Marine20—the marine radiocarbon age calibration curve (0–55,000 cal BP). Radiocarbon. 2020;62(4):779–820.

[85] Petchey F, Spriggs M, Leach F, Seed M, Sand C, Pietrusewsky M, et al. Testing the human factor: radiocarbon dating the first peoples of the South Pacific. Journal of Archaeological Science. 2011;38: 29–44.

[86] Petchey F, Spriggs M, Bedford M, Valentin F, Buckley HR. Radiocarbon dating of burials from the Teouma Lapita cemetery, Efate, Vanuatu. Journal of Archaeological Science. 2014;50:227–42.

[87] Reimer P, Reimer R. A Marine reservoir correction database and on-line interface. Radiocarbon. 2001;43:461–3.

[88] Ledermann S. Nouvelles tables-types de mortalité. Paris: Presses Universitaires de France; 1969. (Travaux et Documents; vol. 53).

[89] Sellier P. Paléodémographie et archéologie funéraire: les cimetières de Mehrgarh, Pakistan. Paléorient. 1995;21(2):123–43.

[90] Sellier P. La mise en évidence d’anomalies démographiques et leur interprétation: population, recrutement et pratiques funéraires du tumulus de Courtesoult. In: Piningre JF, editor. Nécropoles et Société au premier âge du Fer: le tumulus de Courtesoult (Haute-Saône). Paris: Edition de la Maison des Sciences de l’Homme; 1996. p. 188–202. (DAF).

[91] Kacki S. Influence de l’état sanitaire des populations anciennes sur la mortalité en temps de peste: contribution à la paléoépidémiologie. PhD thesis, University of Bordeaux. 2016.

[92] Castex D, Kacki S. Demographic patterns distinctive of epidemic cemeteries in archaeological samples. Microbiology Spectrum. 2016;4(4): 10.1128. doi: 10.1128/microbiolspec.PoH-0015-2015 27726822

[93] de Lépinau AD, Castex D, Brzobohatá H, Frolík J, Velímský F, Brůžek J, et al. Entre peste et famine: caractérisation d’une crise de mortalité par l’étude de trois sépultures multiples du site de Kutná Hora-Sedlec (République tchèque, XIVe siècle). Bulletins et mémoires de la Société d’Anthropologie de Paris. 2021;33(2).

[94] Hedges R, Clement JG, David C, Thomas L, O’Connell TC. Collagen turnover in the adult femoral mid-shaft: modeled from anthropogenic radiocarbon tracer measurements. American Journal of Physical Anthropology. 2007;133:808–16. doi: 10.1002/ajpa.20598 17405135

[95] AlQahtani SJ, Hector MP, Liversidge HM. Brief communication: The London atlas of human tooth development and eruption. American Journal of Physical Anthropology. 2010;142(3):481–90. doi: 10.1002/ajpa.21258 20310064

[96] Zinger W. Variations morphologiques mandibulaire des sociétés actuelles et anciennes d’Océanie et d’Asie du sud-est. Master Thesis, Muséum National d’Histoire Naturelle. 2017.

[97] Nunn PD, Ishimura T, Dickinson WR, Katayama K, Thomas F, Kumar R, et al. The Lapita Occupation at Naitabale, Moturiki Island, Central Fiji. Asian Perspectives. 2007;46:96–132.

[98] Valentin F, Sand C, Le Goff I, Bocherens H. An early first millennium AD burial from the Naselala site, Cikobia-i-Ra Island (North-East Fiji). In: Addison DJ, Asaua TS, Sand C, editors. Recent Archaeology in the Fiji/West-Polynesia Region, Papers from the Archaeology of the Polynesian Homeland Conference. Dunedin: Otago University Press, Studies in Prehistoric Anthropology; 2008. p. 45–56.

[99] Ambrose SH. Preparation and characterization of bone and tooth collagen for isotopic analysis. Journal of Archaeological Science. 1990;17:431–51.

[100] Fitzpatrick SM, Jew N. Radiocarbon dating and Bayesian modelling of one of Remote Oceania’s oldest cemeteries at Chelechol ra Orrak, Palau. Antiquity. 2018;92(361):149–64.

[101] Bedford S, Buckley H, Valentin F, Tayles N, Longga N. Lapita burials, a new Lapita cemetery and post-Lapita burials from Malakula, Northern Vanuatu, Southwest Pacific. Journal of Pacific Archaeology. 2011;2(2):26–48.

[102] Pietrusewsky M, Galipaud JC, Leach F. A skeleton from the Lapita site at Koné, Foué Peninsula, New Caledonia. New Zealand Journal of Archaeology. 1998;1996:25–74.

[103] Valentin F. Human skeletal remains from the site of Lapita at Koné (New Caledonia): mortuary and biological features. In: Sand C, editor. Pacific Archaeology: assessments and prospects. Nouméa: Service des Musées et du Patrimoine; 2003. p. 285–93. (Les Cahiers de l’Archéologie en Nouvelle-Calédonie).

[104] Pietrusewsky M, Hunt TL, Ikehara-Quebral RM. A Lapita-associated skeleton from Waya island, Fiji. Micronesica. 1997;30(2):355–88.

[105] Valentin F, Clark G, Parton P, Reepmeyer C. Mortuary practices of the first Polynesians: formative ethnogenesis in the Kingdom of Tonga. Antiquity. 2020;94(376):999–1014.

[106] Clark G, Grono E, Ussher E, Reepmeyer C. Early settlement and subsistence on Tongatapu, Kingdon of Tonga: insights from a 2700–2650 cal BP midden deposit. Journal of Archaeological Science: Reports. 2015;3:513–24.

[107] Kinaston RL, Bedford SB, Spriggs M, Anson D, Buckley H. Is there a ‘Lapita diet’? A comparison of Lapita and post-Lapita skeletal samples from four Pacific island archaeological sites. In: Oxenham M, Buckley H, editors. The Routledge Handbook of Bioarchaeology in Southeast Asia and the Pacific. Abingdon: Taylor & Francis Group; 2016. p. 427–61.

[108] O’Connell TC, Kneale CJ, Tasevska N, Kuhnle GGC. The diet-body offset in human nitrogen isotopic values: A controlled dietary study. American Journal of Physical Anthropology. 2012;149(3):426–34. doi: 10.1002/ajpa.22140 23042579 PMC3483624

[109] Team RC. R vesion 3.6.1 (R foundation for Statistical Computing). Vienna, Austria; 2018.

[110] Benjamini Y, Yekutieli D. The control of the false discovery rate in multiple testing under dependency. The Annals of Statistics. 2001;29(4):1165–88.

[111] Margerison BJ, Knüsel CJ. Paleodemographic comparison of a catastrophic and an attritional death assemblage. American Journal of Physical Anthropology. 2002;119(2):134–43. doi: 10.1002/ajpa.10082 12237934

[112] Poulsen J. Early Tongan prehistory. Volumes 1 and 2. Terra Australis 12. Canberra: Department of Prehistory, Research School of Pacific Studies; 1987.

[113] Sand C, Ouetcho A, Bolé J, Baret D, Lagarde L, Valentin F. Archéologie en Mélanésie: données du site côtier WBR047 des “Ecrins de Poé” (Bourail, Nouvelle-Calédonie). Bulletin de la Société Préhistorique Française. 2012;109(3):495–512.

[114] Valentin F. Ecrin de Poé II, Inventaire des faits funéraires fouillés en 2008. Paris: CNRS UMR 7041; 2009. (IANCP, Nouméa).

[115] Valentin F. Ecrin de Poé II. Rapport préliminaire sur l’étude archéo-anthropologique des faits funéraires fouillés en 2008. Paris: CNRS UMR 7041; 2009. (IANCP, Nouméa).

[116] Beaumont J, Atkins EC, Buckberry J, Haydock H, Horne P, Howcroft R, et al. Comparing apples and oranges: Why infant bone collagen may not reflect dietary intake in the same way as dentine collagen. Vol. 167, American Journal of Physical Anthropology. 2018. p. 524–40. doi: 10.1002/ajpa.23682 30187451 PMC6221104

[117] Walter BS, DeWitte SN, Dupras T, Beaumont J. Assessment of nutritional stress in famine burials using stable isotope analysis. American Journal of Physical Anthropology. 2020;172(2):214–26. doi: 10.1002/ajpa.24054 32243588

[118] Seemann B. Viti: an account of a government mission to the Vitian or Fijian Islands, in the years 1860–61. London: Macmillan & co; 1862. (1rst Edition).

[119] Sahlins MD. Moala: Culture and Nature on a Fijian Island. University of Michigan Press; 1962.

[120] Williams T. Fiji and the Fijians: The islands and their inhabitants. Suva: Fiji Museum; 1982.

[121] Jones S. Food and gender in Fiji: ethnoarchaeological explorations. Lanham (MD): Lexington Books; 2009.

[122] Roos CI, Field JS, Dudgeon JV. Anthropogenic burning, agricultural intensification, and landscape transformation in Post-Lapita Fiji. Journal of Ethnobiology. 2016;36(3):535–53.

[123] Valentin F, Détroit F, Sprigg MJT, Bedford S. Early Lapita skeletons from Vanuatu show Polynesian craniofacial shape: Implications for Remote Oceanic settlement and Lapita origins. Proceedings of the National Academy of Sciences of the United States of America. 2016;113(2):292–7. doi: 10.1073/pnas.1516186113 26712019 PMC4720332

[124] Lipson M, Skoglund P, Spriggs M, Valentin F, Bedford S, Shing R, et al. Population Turnover in Remote Oceania Shortly After Initial Settlement. Current Biology. 2018;28(7):1157–65. doi: 10.1016/j.cub.2018.02.051 29501328 PMC5882562

[125] Posth C, Nägele K, Colleran H, Valentin F, Bedford S, Kami KW, et al. Waves of history in Remote Oceania: complex population replacement with language continuity in Vanuatu. Nature Ecology and Evolution. 2018;2(4):731–40. 29487365 10.1038/s41559-018-0498-2 PMC5868730

[126] Zinger W. L’inconnu derrière la vague. Contribution à l’histoire des migrations polynésiennes en Mélanésie Australe: études exploratoires des variations phénotypiques sur 3000 ans d’histoire. PhD Thesis, Museum National d’Histoire Naturelle. 2021.

[127] Clark JT, Cole AO, Nunn PD. Environmental change and human prehistory on Totoya island, Fiji. In: Galipaud JC, Lilley I, editors. The Pacific from 5000 to 2000 BP: Colonizations and Transformations. Paris: Editions de l’IRD; 1999. p. 227–40.

[128] Cochrane EE, Rivera-Collazo IC, Walsh E. New evidence for variation in colonization, cultural transmission, and subsistence from Lapita (2900 BP) to the Historic period in Southwestern Fiji. Journal of Pacific Archaeology. 2011;2(1):40–55.

[129] Sand C, Valentin F. Pacific prehistory: an example of cultural evolution from the island of Cikobia (Macuata, Fiji). In: Waldren WH, Ensenyat JA, editors. World Islands in Prehistory: international insular investigations. BAR International Series. 2002. p. 91–7.

