# Supplementary material for: Life histories in Fiji as reconstructed from first millennium CE Sigatoka Sand Dune burials using isotopes
Source: PLoS One. 2024 May 9;19(5):e0300749. doi: 10.1371/journal.pone.0300749 (PMC11081393; doi:10.1371/journal.pone.0300749)
Supplement: S1 Text — (DOCX) [file pone.0300749.s005.docx]

**Life histories in Fiji as reconstructed from first millennium CE Sigatoka Sand Dune burials using isotopes**

**Estelle Herrscher^1^, Frédérique Valentin^2^, Wanda Zinger^3^, Baptiste Pradier^2^, Guy André^1^**

**1** Aix Marseille Univ, CNRS, Minist Culture, LAMPEA, Aix–en–Provence, France, **2** UMR 8068, TEMPS, MSH Mondes, Nanterre, France, **3** Archaeo-and Palaeogenetics group, Institute for Archaeological Sciences, University of Tübingen, Germany

**S1 Text. Supporting information for the stable isotope analysis**

38 bone and 30 teeth were sampled at Fiji Museum. All samples were prepared at the biochemical Laboratory at UMR 7269 LAMPEA, Aix-en-Provence (France). The extracted collagens were sent to Iso-Analytical Limited (U.K.) for analysis. Due to the poor macroscopic preservation of bones and teeth, a gentle method of collagen extraction was preferred as those proposed by [1] on whole chunk rather than the modified Longin method powdered-based [2]. Chunk samples of bone and dentin are submerged in a dilute HCl solution (0.5 N) at 4°C until samples were fully demineralized. Bone and dentine samples were then removed from the acid solution, rinsed with MiliQ until to get a clear solution, then submerged in a 0.125N solution of NaOH at room temperature for less than 15 hours. After several rinsing bath in MiliQ samples are solubilized in HCl solution (pH3, 75°C, 48hrs). The resultant soluble collagen was filtered with Ezee filters and subsequently lyophilized.

Samples were measured with a Europa Scientific^TM^ elemental analyzer, coupled to a mass spectrometer at Isoanalytical (Crewshire, UK). Carbon and nitrogen isotope values are reported in “per mil (‰)” and are calibrated to VPDB and AIR, respectively, using IA–R068. IA–R038, IA–R069, and a mixture of IA–R046 and IAEA–C7 are used as control check standards. IA–R068, IA–R038, and IA–R069 are calibrated and traceable to the international standards IAEA–CH–6 and IAEA–N–1. IA–R046 is calibrated against and traceable to the international standards IAEA–N–1 and IAEA–C7. We calculated the total analytical uncertainties for *δ*^13^C and *δ*^15^N values following the recommendations of Szpak [3]. These analytical uncertainties integrate the precision of the measurements based on the reproducibility of the measurements of a repeated sample (±0.1 ‰ for *δ*^13^C and *δ*^15^N) and the systematic errors based on the repeated analyzes of the check standards (±0.15 ‰ for *δ*^13^C and ±0.20 ‰ for *δ*^15^N). In this study, the difference between 2 isotope ratios is only considered significant if it is greater than ±0.4 ‰.

The quality of the collagen is assessed using the conventional criteria: collagen extraction yield >10mg.g-1, %C between 15.3 % and 47 %, %N between 5.5 and 17.3 %, atomic C/N ratio between 2.9 and 3.6 [4,5,6]. Only samples with elemental compositions within these ranges are accepted for analysis.

**References**

1. Sealy J. Stable carbon isotopes and Prehistoric diets in the Southwestern Cape Province, South Africa. *BAR International Series.* 1986 293:150.
2. Herrscher E, Fenner JN, Valentin F, et al. Multi-isotopic analysis of first Polynesian diet (Talasiu, Tongatapu, Kingdom of Tonga). *Journal of Archaeological Science: Reports.* 2018;18:308-317.
3. Szpak P, Metcalfe JZ, and Macdonald RA. 2017. Best practices for calibrating and reporting stable isotope measurements in archaeology. *Journal of Archaeological Science: Reports* 13:609-616.
4. Ambrose SH. Preparation and characterization of bone and tooth collagen for isotopic analysis. *Journal of Archaeological Science.* 1990;17:431-451.
5. DeNiro MJ. Post-mortem preservation and alteration of in vivo bone collagen isotope ratios in relation to paleodietary reconstruction. *Nature.* 6032. 1985;317:806-809.
6. van Klinken GJ. Bone collagen quality indicators for palaeodietary and radiocarbon measurements. *Journal of Archaeological Science.* 1999;26:687-695.
